# Electroacupuncture prevents endothelial dysfunction induced by ischemia-reperfusion injury via a cyclooxygenase-2-dependent mechanism: A randomized controlled crossover trial

**DOI:** 10.1371/journal.pone.0178838

**Published:** 2017-06-07

**Authors:** Seung Min Kathy Lee, Hyun Soo Kim, Jimin Park, Jong Shin Woo, Jungtae Leem, Jun Hyeong Park, Sanghoon Lee, Hyemoon Chung, Jung Myung Lee, Jin-Bae Kim, Woo-Shik Kim, Kwon Sam Kim, Weon Kim

**Affiliations:** 1 Department of Clinical Korean Medicine, Graduate School, Kyung Hee University, Seoul, Republic of Korea; 2 East-West Medical Research Institute, Kyung Hee University, Seoul, Republic of Korea; 3 Department of Acupuncture and Moxibustion, College of Korean Medicine, Kyung Hee University, Seoul, Republic of Korea; 4 Division of Cardiology, Department of Internal Medicine, Kyung Hee University Hospital, Kyung Hee University, Seoul, Republic of Korea; Stanford University School of Medicine, UNITED STATES

## Abstract

**Objective:**

Exploring clinically effective methods to reduce ischemia-reperfusion (IR) injury in humans is critical. Several drugs have shown protective effects, but studies using other interventions have been rare. Electroacupuncture (EA) has induced similar protection in several animal studies but no study has investigated how the effects could be translated and reproduced in humans. This study aimed to explore the potential effect and mechanisms of EA in IR-induced endothelial dysfunction in humans.

**Methods:**

This is a prospective, randomized, crossover, sham-controlled trial consisting of two protocols. Protocol 1 was a crossover study to investigate the effect of EA on IR-induced endothelial dysfunction. Twenty healthy volunteers were randomly assigned to EA or sham EA (sham). Flow mediated dilation (FMD) of the brachial artery (BA), nitroglycerin-mediated endothelial independent dilation, blood pressure before and after IR were measured. In protocol 2, seven volunteers were administered COX-2 inhibitor celecoxib (200 mg orally twice daily) for five days. After consumption, volunteers underwent FMD before and after IR identical to protocol 1.

**Results:**

In protocol 1, baseline BA diameter, Pre-IR BA diameter and FMD were similar between the two groups (p = NS). After IR, sham group showed significantly blunted FMD (Pre-IR: 11.41 ± 3.10%, Post-IR: 4.49 ± 2.04%, p < 0.001). However, EA protected this blunted FMD (Pre-IR: 10.96 ± 5.30%, Post-IR: 9.47 ± 5.23%, p = NS, p < 0.05 compared with sham EA after IR). In protocol 2, this protective effect was completely abolished by pre-treatment with celecoxib (Pre-IR: 11.05 ± 3.27%; Post-IR: 4.20 ± 1.68%, p = 0.001).

**Conclusion:**

EA may prevent IR-induced endothelial dysfunction via a COX-2 dependent mechanism.

## Introduction

Previous studies have emphasized the importance of vascular endothelial cells, which are particularly susceptible to and actively participate in the pathophysiology of tissue injury induced by ischemia-reperfusion (IR). In the clinic, the chronic dysfunction of these endothelial cells leads to atherosclerosis and also acts as an important independent predictor of negative prognosis for coronary artery diseases [1]. As it can act as a determinant factor reflecting a tissue’s capacity to recover from IR, interventions to protect the endothelium from IR has become an area of great clinical interest. In a human forearm IR model, both exposure to brief periods of ischemia (ischemic preconditioning) [2] and some pharmacologic stimuli such as sildenafil, rosuvastatin and exenatide have been shown to reduce IR-induced endothelial dysfunction [3–5].

In pharmaceuticals, several molecules have been identified as mediators in maintaining endothelial function. Nitric oxide (NO) is considered one of the most significant as it is known to be involved in several of the protective mechanisms [6], including the up-regulation of the cyclooxygenase (COX)-2 enzyme [7–9]. Studies show that prostaglandins mediate the cardioprotective effects of atorvastatin, 3-hydroxy-3-methylglutaryl coenzyme A (HMG-CoA) reductase inhibitors, against IR injury [7]. Rosuvastatin has also been demonstrated to induce such a preconditioning response mediated through a COX-2-dependent mechanism [4].

In complementary and alternative medicine, acupuncture is commonly used to alleviate pain but it has also been widely explored in the treatment of hypertension [10] and many cardiovascular diseases [11–14]. Acupuncture has been reported to reduce sympathetic tone and enhance the generation of endothelial NO [15]. Animal studies in hypertensive models have also shown that acupuncture, especially electroacupuncture (EA), works via stimulation of sensory nerve fibers that decrease sympathetic outflow and norepinephrine release, while increasing c-fos expression in the nucleus of the solitary tract [16]. Yet, the mechanisms at work in prevention of endothelial dysfunction are still under exploration.

A preliminary study in patients with hypertension suggested that acupuncture improved FMD [17], but it did not explore the mechanisms. Since then no other clinical study has investigated how acupuncture protects against IR-induced endothelial dysfunction in a human model. Therefore we designed two protocols to test (1) whether acupuncture can prevent impairment in IR-induced endothelial dysfunction and (2) whether this effect is mediated by a COX-2 dependent pathway in a human forearm model of IR-induced endothelial dysfunction.

## Methods

### 2.1 Patient population

**Fig 1:**
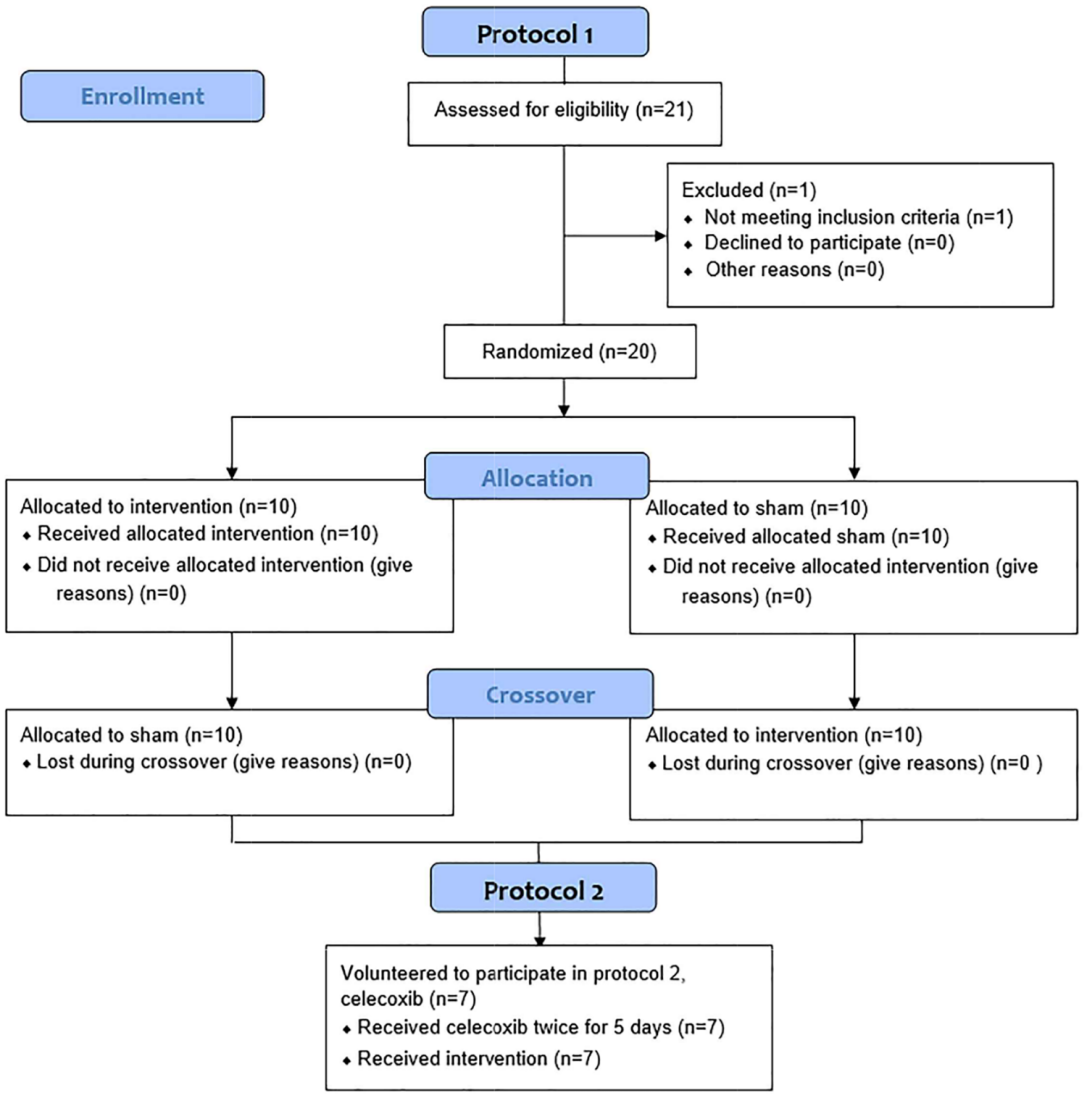
Flow diagram. Consolidated standards of reporting trials flow diagram of the randomized controlled trial.

From May 1^st^ to August 23^rd^ 2014, healthy nonsmoking volunteers (25 to 40 years of age) were recruited and those currently taking medication for hypertension, diabetes or other chronic diseases, suffering from hepatic, renal, thyroid, or cerebrovascular diseases, currently pregnant, or has body mass index greater than 25 kg/m^2^ were excluded. After trial commencement, we further excluded those taking dietary supplements or enrolled in another clinical trial. Twenty one people were assessed for eligibility and twenty people fulfilled the inclusion criteria. All of them completed two sessions of treatment for protocol 1. Among the participants, seven volunteered to further take part in the celebrex experimental group (protocol 2) (Fig 1).

### 2.2 Study design

This study was a prospective, randomized, subject and assessor blind, crossover and placebo-controlled clinical trial conducted in the Division of Cardiology, Department of Internal Medicine, and the Department of Acupuncture and Moxibustion, Kyung Hee University Medical Centre, Seoul, Republic of Korea. Data were collected in the Division of Cardiology, Kyung Hee University Medical Centre. This study protocol was approved by the institutional review board (IRB) of Kyung Hee University Hospital (approval date: April 11^th^, 2014, No. 1403–01) and was developed in accordance with the Declaration of Helsinki. The study protocol was explained in detail to eligible participants, and written informed consent was obtained from all subjects. ₩ 100,000 (approximately $ 90) was given to each participant per session as compensation for their time. The study was registered at Clinical Trials.gov: NCT02255006 (https://clinicaltrials.gov/ct2/show/NCT02255006). There was a delay in registering this study due to language and administrative issues. The authors confirm that all ongoing and related trials for this intervention are registered.

#### 2.2.1. Protocol 1: Effect of electroacupuncture (EA) on IR-induced endothelial dysfunction

**Fig 2:**
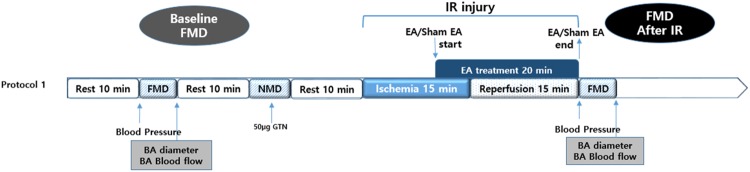
Study protocol. Ischemia-reperfusion and flow-mediated dilatation were measured and subjects received electroacupuncture or sham electroacupuncture according to the assigned sequence of treatment phases. BA: basal artery, EA: electroacupuncture, FMD: flow-mediated dilatation, IR: ischemia-reperfusion, NMD: nitroglycerin-mediated endothelial independent dilation.

Protocol 1 was performed by a randomized, subject and assessor blind, crossover and placebo-controlled manner. Twenty healthy nonsmoking volunteers (9 males; ages 29.6 ± 2.6 years) were enrolled in the trial. All subjects were asked to abstain from caffeine and drugs, including supplemental vitamins, for the duration of the study. Initially, endothelium-dependent FMD of the brachial artery (BA) and nitroglycerin-mediated endothelial independent dilation (NMD) were measured before and after IR (15 minutes of ischemia at the level of the proximal upper arm followed by 15 minutes of reperfusion) (Fig 2). EA was performed beginning 10 minutes after ischemia to the end of reperfusion (20 minutes total). All subjects underwent a seven-day wash-out period. Seven days later, the subjects returned to participate in the crossover study (i.e., EA or sham EA), and the protocol described above was repeated. Supine blood pressure (BP) was taken and recorded before the exam and at the end of the exam described in protocol 1.

#### 2.2.2 Randomization and blinding

Participants were randomly assigned to either EA or sham EA using a computerized random allocation sequence with a permutable block design. A member who had no contact with any of the participants notified only the acupuncturist of the participant allocation right before treatment by phone. Assessors were concealed to all of the allocations, and the participants were naturally blinded because the point of real EA and sham EA were very close, the study was conducted in a semi-lit evaluation room limiting view of the treatment area, and most were acupuncture naïve. The acupuncturists only came into the study room to insert the needles and to apply EA while refraining from any conversation.

#### 2.2.3 Protocol 2: Effect of celecoxib pre-treatment

Protocol 2 was performed in a non-randomized, non-blinded manner. After completing protocol 1, seven volunteers were further recruited to participate in protocol 2. They underwent a fifteen-day wash-out period. Volunteers were administered the selective COX-2 inhibitor, celecoxib (Cerebrex, Pfizer) 200 mg twice a day for five days. The last dose of celecoxib was administered on the morning of FMD measurement. Subjects underwent FMD measurement before and after IR, as described above. EA was performed for 20 minutes as in the previously described method.

#### 2.2.4 EA and sham EA

For active EA treatment, unilateral PC5, PC6, ST36, and ST37 were used (S1 Fig). PC5 and PC6 are located proximal to the base of the palmar wrist crease above the median nerve. ST36 and ST37 are located below the depression point lateral to the patellar ligament and lateral to the anterior tubercle of the tibia on the anterior aspect of the knee. PC5 and ST37 were added to connect the poles to PC6 and ST36. Previous clinical studies have consistently shown that acupuncture stimulation on PC6 plus ST36 improves endothelial function [17,18].

Disposable, sterile needles (0.25 mm x 40 mm, Dongbang Acupuncture Inc., Boryung, Korea) and a low-frequency electrical stimulator (ES-160, ITO, Japan) were used. Each needle was inserted at a 90 degree angle to a depth of 1cm at PC5 and PC6 and to a depth of 2 cm at ST36 and ST37. Then, the practitioner rotated the needle several times and induced de-qi (acupuncture-evoked sensations such as heaviness, numbness, soreness, or distention). Thereafter, needles were connected to the pole and electrical stimulation was applied with a 2 Hz continuous wave current. Intensity was increased until participants felt stimulation but not discomfort, and their muscles twitched slightly. EA was performed after ten minutes of inducing ischemia to the end of reperfusion, 20 minutes in total. Acupuncture treatment was conducted by Doctors of Korean Medicine educated by the College of Korean Medicine for six years and licensed by the Korean Ministry of Health and Welfare; with more than four years of clinical experience. All practitioners were trained to follow the study protocol.

For sham EA treatment, four non-acupuncture points (located 10–20 mm lateral to PC5, PC6, ST36, ST37 and not above a meridian line) were inserted to a depth of 0.5 cm at a 90 degree angle. Manual manipulation was not conducted, and de-qi sensations were not induced. Needles were connected to the pole and identical sounds were generated as in the active EA group, but electrical stimulation was not delivered to the sham EA group. After each session, any bleeding, bruises, or unintended effects due to EA were identified and documented. These EA and sham EA methods have been used successfully in previous clinical trials [17, 18].

#### 2.2.5 Measurement of FMD

Subjects were asked to rest for ten minutes in the supine position prior to each study protocol. The FMD and NMD measurements were performed by two independent operators. Brachial artery images were obtained using a commercially available system (Vivid 7, GE Vingmed, Horten, Norway) equipped with a 14-MHz linear array transducer. The ECG-gated, end-diastolic, longitudinal, B-mode images were digitally stored on the hard disk of the instrument for on-line and off-line analysis. For FMD measurement, the baseline BA diameter was averaged from six separate images taken at five-second intervals. Subsequently, a pneumatic cuff placed at the level of the lower arm was inflated to 200 mmHg for five minutes. After wrist-cuff deflation, the BA diameter was re-examined and averaged from six separate images taken at five-second intervals. FMD was calculated as the percent maximum increase in arterial diameter. After a ten-minute rest period to allow restoration of baseline conditions, NMD was again assessed by obtaining two-dimensional images before and three minutes after administration of 50 μg of GTN in order to characterize endothelial-independent vasodilation. The artery diameter was calculated from the trailing edge of the intima-blood interface to the leading edge semi-automatically using a modified version of Image J software (Version 1.47 t, National Institutes of Health, Bethesda, MD, USA) as well as custom-designed software. Baseline and post-IR (reactive hyperemia) blood flows were measured using pulsed-wave Doppler as an average velocity-time integral for the first five cardiac cycles after cuff deflation and were multiplied by heart rate and vessel cross-sectional area. The reproducibility of this method in our laboratory was previously reported several times [5,19,20]. A diagram illustrating the study method is shown in S2 Fig.

### 2.3 Statistical analysis

Sample size was calculated as follows. In our preceding research among healthy subjects, FMD before IR injury was 12.0 ± 6.2, and after IR was 4.6 ± 3.6 [5]. Exenatide was proven to prevent IR injury 100% (FMD before IR = 15.0 ± 7.1, FMD after IR = 15.0 ± 5.9). Yet, the prevention effect of acupuncture has not been studied. We assumed that acupuncture could improve FMD after IR injury about 30%. Targeted number of subjects is total 34 (17 in each group), considering 5% type 1 error and 80% power. Considering drop-out rate of 15%, total 40 subjects are needed. As this study is a crossover trial, 20 subjects are needed. Data were presented as the mean ± standard deviation. Within-group comparisons were performed with paired *t*-test or Wilcoxon signed rank test. Between-group differences and the interaction of IR and group randomization were studied with a two-way analysis of variance. Post hoc comparisons were performed using the Bonferroni correction. A *p*-value less than 0.05 was considered statistically significant. Multivariate analysis (adjusted age and gender) was performed to determine whether acupuncture could protect endothelial dysfunction defined as FMD < 6%. All analyses were performed with SPSS software (Windows version 17.0, Chicago, Illinois, USA) by a statistician blinded to participant allocation. The CONSORT checklist, original, and translated version of the trial protocol are available (S1, S2 and S3 Files).

## Results

### 3.1 Baseline characteristics

All of the eligible participants completed the trial without dropping out. The baseline demographic characteristics of the subjects in protocol 1 and protocol 2 are summarized in Table 1. There were no reported adverse effects. The individual data points behind means, medians and variance measures presented in the results are available (S4 File).

**Table 1:** Baseline demographic characteristics of subjects.

Variables	Protocol 1 Subjects (*n* = 20)	Protocol 2 Subjects (*n* = 7)
Age (yr)	29.6 ± 2.6	28.2 ± 2.3
Male (*n*)	9 (45%)	5 (71%)
Smoker (n)	0	0
Body mass index (kg/m^2^)	20.5 ± 2.4	21.4 ± 1.9
Hemoglobin (g/dL)	14.7 ± 1.0	14.7 ± 1.5
Platelet count (10^3^/μL)	233.3 ± 50.5	248.5 ± 35.5
Aspartate aminotransferase (U/L)	24.8 ± 4.2	21.1 ± 5.2
Alanine aminotransferase (U/L)	19.4 ± 9.3	19.0 ± 8.9
Creatinine (mg/dL)	0.7 ± 0.1	0.7 ± 0.1
Fasting glucose (mg/dL)	91.0 ± 5.8	96.4 ± 11.9
Total cholesterol (mg/dL)	192.6 ± 38.3	177.6 ± 24.3
Triglyceride (my/dL)	83.7 ± 51.1	64.2 ± 17.1
High-density lipoprotein cholesterol (mg/dL)	114.4 ± 34.1	98.6 ± 18.5
Low-density lipoprotein cholesterol (mg/dL)	64.0 ± 13.4	63.7 ± 10.8

### 3.2 Protocol 1

EA intervention had no effect on arterial BP compared with sham EA (systolic BP: EA 113.05 ± 12.99 mmHg vs. sham EA: 113.75 ± 13.26 mmHg, P = NS, diastolic BP: EA 66.6 ± 7.71 mmHg vs. placebo EA 67.4 ± 9.45 mmHg, P = NS). EA and sham EA had no effect on baseline BA diameter before and after IR (Table 2, p = NS).

**Table 2:** Results of arterial diameter in protocols 1 and 2.

	Pre-IR			Post-IR		
	FMD(%)	Baseline (mm)	Diameter after cuff deflation	FMD(%)	Baseline (mm)	Diameter after cuff deflation
**Protocol 1**						
EA	10.96 ± 5.30	3.42 ± 0.55	3.77 ± 0.50	9.47 ± 5.23^#^	3.51 ± 0.60	3.83 ± 0.66
Sham	11.41 ± 3.10	3.41 ± 0.48	3.80 ± 0.54	4.49 ± 2.04*	3.47 ± 0.48	3.63 ± 0.51
**Protocol 2**						
Celecoxib + EA	11.05 ± 3.27	3.43 ± 0.32	3.81 ± 0.36	4.20 ± 1.68*	3.66 ± 0.53	3.81 ± 0.53

Baseline data refer to brachial artery diameter before upper arm cuff occlusion. Values are the mean ± standard deviation. EA: electroacupuncture, FMD: flow-mediated dilatation; IR: ischemia-reperfusion.

*p < 0.05 vs. the corresponding value before IR.

^#^p = 0.002 compared with sham EA.

#### 3.2.1 Effects of IR injury after sham EA

**Fig 3:**
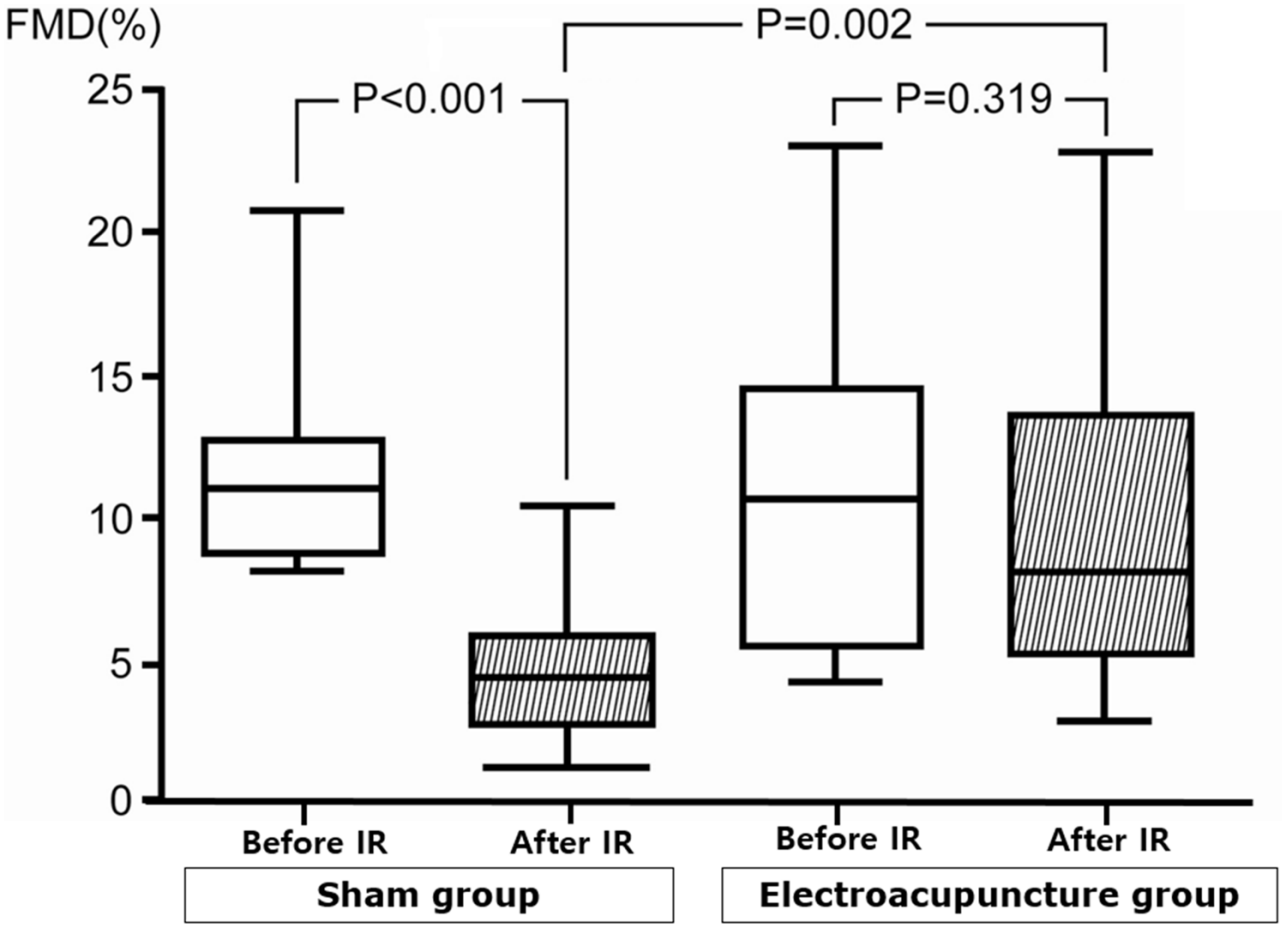
Box plots of the flow-mediated dilatation responses before and after ischemia-reperfusion (protocol 1). Left: In the sham group, FMD was significantly blunted after IR. Right: Electroacupuncture completely prevented the impairment in endothelium-dependent vasodilation induced by IR. Boxes show interquartile ranges; the lower and upper boundaries of the boxes indicate the 25^th^ and 75^th^ percentile levels, respectively, and the horizontal lines within the boxes indicate the median levels. FMD: flow-mediated dilatation, IR: ischemia-reperfusion.

In the sham EA group, BA diameter and blood flow values were not different from baseline values before and after IR (p = NS). However, post-IR FMD was significantly decreased compared with the pre-IR level (Pre-IR: 11.41 ± 3.10%; Post-IR: 4.49 ± 2.04%, p < 0.001, Table 2 and Fig 3). However, there was no significant difference in blood flow (Pre-IR: 237.4 ± 158.5 mL/min; Post-IR: 188.6 ± 87.1 mL/min, p = NS, Table 3) and NMD (pre-IR: 14.22 ± 3.76%; post- IR: 11.53 ± 4.36%, p = NS).

**Table 3:** Results of brachial artery blood flow in protocols 1 and 2.

Blood Flow	Blood Flow Pre-IR		Blood Flow Post-IR	
(mL/min)	Baseline	after cuff deflation	Baseline	after cuff deflation
**Protocol 1**				
EA	74.47 ± 40.2	240.6 ± 148.8	68.9 ± 46.9	204.9 ± 111.1
Sham	77.3 ± 45.9	237.4 ± 158.5	67.5 ± 28.9	188.6 ± 87.1
**Protocol 2**				
Celecoxib + EA	85.6 ± 37.6	242.9 ± 57.6	70.34 ± 15.2	209.2 ± 49.6

Values are the mean ± standard deviation. IR indicates ischemia-reperfusion.

EA: electroacupuncture.

#### 3.2.2 Effects of IR injury after EA

Similarly, BA diameter and blood flow values were not different from baseline values before and after IR (p = NS) in the EA group. In contrast, EA restored the FMD reduction that was observed in the sham EA group after IR injury (pre-IR: 10.96 ± 5.30%; Post-IR: 9.47 ± 5.23%, p = NS, p < 0.05 compared with sham EA after IR; two-way ANOVA, Table 2 and Fig 3), but there was no statistical difference in blood flow (Pre-IR: 240.6±148.8 mL/min; Post-IR: 204.9 ± 111.1 mL/min, p = NS) and NMD (Pre-IR: 13.98 ± 6.04%; Post-IR: 11.30 ± 4.08%, p = NS).

### 3.3 Protocol 2

#### 3.3.1 Effect of celecoxib

**Fig 4:**
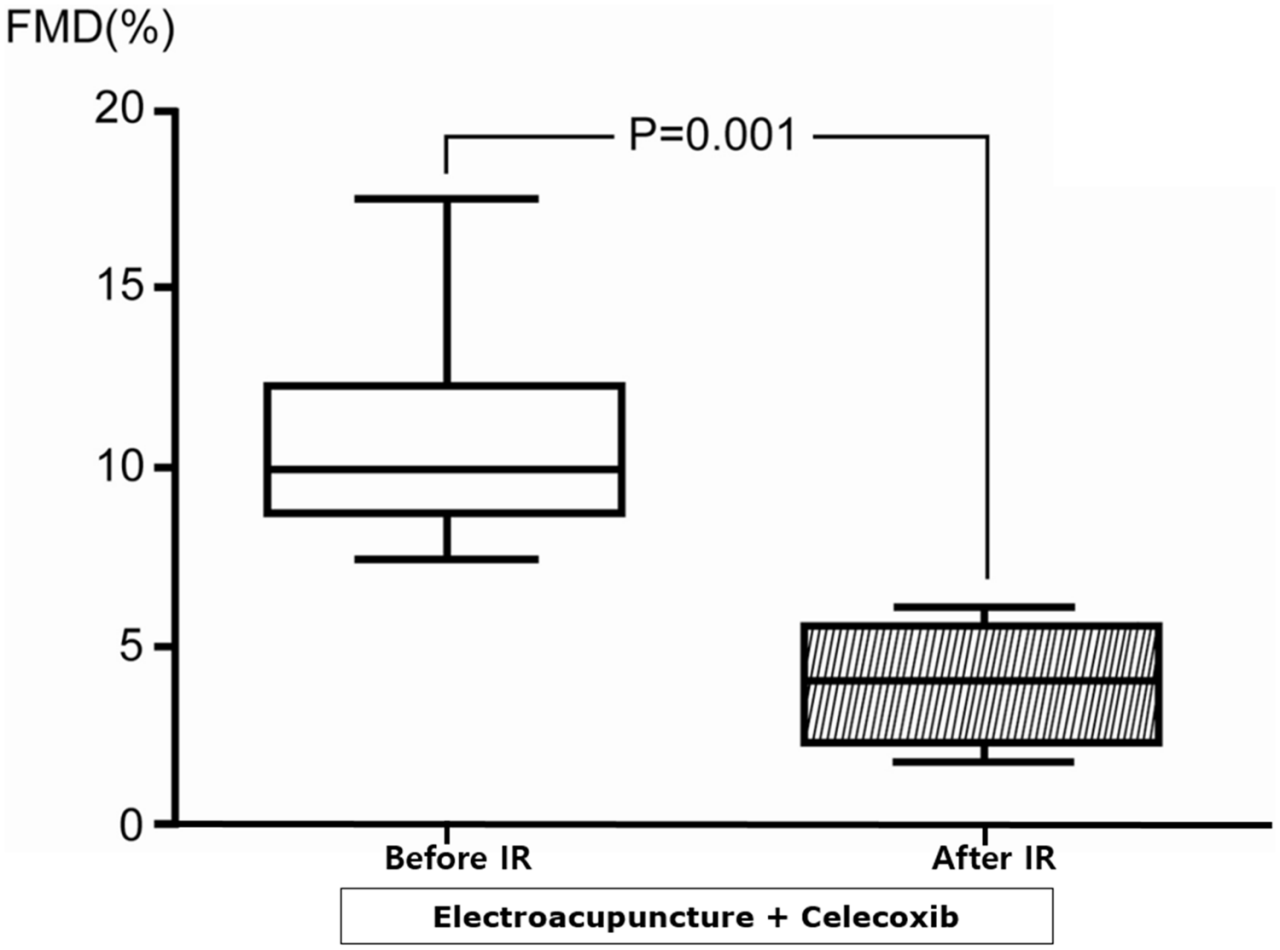
Box plots of the flow-mediated dilatation responses before and after ischemia-reperfusion following administration of celecoxib in the electroacupuncture group (protocol 2).

Pre-IR baseline BA diameter, blood flow, and FMD were not different after celecoxib plus EA compared with EA alone. IR significantly blunted FMD responses in subjects who received celecoxib plus EA (Pre-IR: 11.05 ± 3.27%; Post-IR: 4.20 ± 1.68%, p = 0.001) (Fig 4). IR did not decrease blood flow in the celecoxib plus EA group (pre-IR 242.9 ± 57.6 mL/min, post-IR 209.2 ± 49.6 mL/min, P = NS). FMD was blunted to almost the same values observed in the sham EA group during protocol 1, demonstrating the potent inhibitory effect of celecoxib on EA-induced endothelial protection (EA only 9.47 ± 5.23% vs. celecoxib plus EA 4.20 ± 1.68%, P < 0.05 compared with two-way ANOVA). Multivariate analysis adjusting age and gender determined that sham (HR (hazard ratio) 27.3, 95% CI (confidential interval) 4.6–162.5, P < 0.001) and celecoxib (HR 14.6, 95% CI 1.3–160.3, P = 0.003) treatment were associated with endothelial dysfunction defined as FMD < 6%.

FMD was significantly blunted after IR, demonstrating that celecoxib prevented electroacupuncture-induced endothelial protection against IR. Boxes show interquartile ranges; the lower and upper boundaries of the boxes indicate the 25^th^ and 75^th^ percentile levels, respectively; and the horizontal lines within the boxes indicate the median levels. FMD: flow-mediated dilatation, IR: ischemia-reperfusion.

## Discussion

To the best of our knowledge, this study is the first to demonstrate the protective effects of EA against endothelial dysfunction and to verify the mechanistic significance of COX-2 in a human forearm IR model. We observed a significant blunting of FMD after IR in the sham control group, whereas EA prevented it. We observed a significant blunting of FMD after IR in the sham control group, whereas EA prevented it. Since EA treatment was performed on the contralateral side of IR injury, it also revealed that a big part of its mechanism relied on central pathways.

Previous animal studies have suggested that the mechanisms leading to such preconditioning—mimetic properties of EA is likely multifactorial, with modulation of the autonomic nervous system (ANS), endothelial nitric oxide synthase (eNOS), and inducible NOS all coming to play [15,21,22]. Especially in the treatment of cardiovascular diseases, modulation of the ANS by suppression of the sympathetic nervous system is acknowledged as one of the central therapeutic mechanisms of EA [16]. Studies have also shown that acupuncture needling on ST36, a point that was used in our trial as well, activates the synthesis of NO [15], which is the main modulator of local blood flow and vascular tone [23]. In IR injury animal models, acupuncture reduced infarct size via the general RISK pathway including anti-apoptotic effects through modulation of the PI3K/Akt pathway [24], and in the cortex, EA reduced infarct size by augmenting eNOS activity thereby increasing cerebral perfusion [25].

In non-acupuncture related clinical studies, the proposed endothelial protection mechanism has also emphasized the role of NO in the physiology of both ischemic and pharmacological preconditioning via a direct and cGMP-mediated opening of KATP channels [26,27]. In endothelium-dependent vasodilation induced by IR injury, we had previously shown that administration of exenatide can prevent impairment and that this effect is mediated by KATP channel opening [5]. Andrew et al. also showed that rosuvastatin could prevent IR-induced conduit artery endothelial dysfunction and that this effect was regulated by a COX-2–dependent mechanism [4]. In 2010, a preliminary study in hypertensive patients suggested that 15 minutes of acupuncture could improve FMD and we saw similar effects using 20 minutes of EA in stroke patients [17, 18].

To further clarify the proposed mechanisms, specific inhibitors are utilized before inducing ischemia. Past studies have revealed that intravenous PG I2 given before ischemia reduced infarct size and administration of COX-2 inhibitors before infarction hindered the infarct size-limiting effects of late ischemic preconditioning [28]. Administration of iNOS inhibitor 24 hours after ischemic preconditioning inhibited the increased production of myocardial PGE2 and 6-keto-PGF1a, while administration of COX-2 inhibitors at the same period did not affect iNOS activity [29,30].

Adding to the current field of knowledge, our study newly demonstrated that the endothelial protection afforded by EA disappears after administration of celecoxib, a COX-2 inhibitor, and suggests that COX-2 may be involved in the signaling pathway leading to EA-mediated protection in a human forearm IR model. In the area of pain research, where EA has been most actively explored, EA also induced its anti-nociceptive effects by modulation of COX-2 expression in the nervous system related to pain control. In a recent study, EA alleviated mechanical and thermal hypersensitivity after lumbar L5 spinal nerve ligation by inhibiting COX-2 expression in the spinal dorsal horn and this inhibition was as effective as the COX-2 inhibitor celecoxib [31]. Along these lines, we think that EA might induce activity similar to that of COX-2 by increasing the NO bioavailability in IR-induced endothelial dysfunction in a human conduit artery.

### Limitations

Our study had a small sample size and was conducted at a single center. Endothelial dysfunction induced by IR injury was limited to the conduit circulation and our assessment did not include the coronary circulation or distal microcirculation, which are also of importance in clinical IR injury. The data were obtained from a sample of healthy volunteers who likely did not have impaired endothelium-dependent responses. Also, among the volunteers in protocol 2, EA non-responders may have been included. Future studies are necessary to investigate the potential clinical implications in greater detail.

### Conclusions

This pilot trial is the first human evidence verifying the effects of EA on endothelial pharmacological preconditioning and suggests that it may be induced via a COX-2-dependent mechanism.

### Ethics approval

Kyung Hee University Medical Centre Ethics Board.

## Supporting information

S1 Fig Acupuncture points. PC5 & PC6, ST36 & ST37, and sham electroacupuncture points. PC: pericardium, ST: stomach.
(TIF)

S2 Fig Diagram of flow-mediated dilatation measurement. Diagram showing how electroacupuncure was conducted and how flow-mediated dilatation was measured on subjects. FMD: flow-mediated dilatation, IR: ischemia-reperfusion.
(TIF)

S1 File CONSORT checklist. Consolidated Standards of Reporting Trials checklist.
(DOC)

S2 File Original trial protocol. Original Korean version of trial protocol.
(DOC)

S3 File Translated trial protocol. Translated version of original trial protocol.
(DOCX)

S4 File Individual data points. Individual data points of the study.
(XLSX)

## References

[1] Widlansky ME, Gokce N, Keaney JF Jr, Vita JA. The clinical implications of endothelial dysfunction. J Am Coll Cardiol. 2003;42:1149–60. 14522472 10.1016/s0735-1097(03)00994-x

[2] Laude K, Beauchamp P, Thuillez C, Richard V. Endothelial protective effects of preconditioning. Cardiovasc Res. 2002;55:466–73. 12160943 10.1016/s0008-6363(02)00277-8

[3] Gori T, Sicuro S, Dragoni S, Donati G, Forconi S, Parker JD. Sildenafil prevents endothelial dysfunction induced by ischemia and reperfusion via opening of adenosine triphosphate-sensitive potassium channels: A human in vivo study. Circulation. 2005;111:742–6. 10.1161/01.CIR.0000155252.23933.2D 15699265

[4] Liuni A, Luca MC, Gori T, Parker JD. Rosuvastatin prevents conduit artery endothelial dysfunction induced by ischemia and reperfusion by a cyclooxygenase-2-dependent mechanism. J Am Coll Cardiol. 2010;55:1002–6. 10.1016/j.jacc.2009.11.046 20202516

[5] Ha SJ, Kim W, Woo JS, Kim JB, Kim SJ, Kim WS, et al Preventive effects of exenatide on endothelial dysfunction induced by ischemia-reperfusion injury via katp channels. Arterioscler Thromb Vasc Biol. 2012;32:474–80. 10.1161/ATVBAHA.110.222653 22155457

[6] Joannides R, Haefeli WE, Linder L, Richard V, Bakkali EH, Thuillez C et al Nitric oxide is responsible for flow-dependent dilatation of human peripheral conduit arteries in vivo. Circulation. 1995;91:1314–9. 7867167 10.1161/01.cir.91.5.1314

[7] Birnbaum Y, Ye Y, Rosanio S, Tavackoli S, Hu ZY, Schwarz ER, et al Prostaglandins mediate the cardioprotective effects of atorvastatin against ischemia-reperfusion injury. Cardiovasc Res. 2005;65:345–55. 10.1016/j.cardiores.2004.10.018 15639473

[8] Atar S, Ye Y, Lin Y, Freeberg SY, Nishi SP, Rosanio S, et al Atorvastatin-induced cardioprotection is mediated by increasing inducible nitric oxide synthase and consequent S-nitrosylation of cyclooxygenase-2. Am J Physiol Heart Circ Physiol. 2006;290:H1960–8. 10.1152/ajpheart.01137.2005 16339820

[9] Ye Y, Martinez JD, Perez-Polo RJ, Lin Y, Uretsky BF, Birnbaum Y. The role of eNOS, iNOS, and NF-kappaB in upregulation and activation of cyclooxygenase-2 and infarct size reduction by atorvastatin. Am J Physiol Heart Circ Physiol. 2008;295:H343–51. 10.1152/ajpheart.01350.2007 18469150

[10] Wang J, Xiong X, Liu W. Acupuncture for essential hypertension. Int J Cardiol. 2013;169:317–26. 10.1016/j.ijcard.2013.09.001 24060112

[11] Lomuscio A, Belletti S, Battezzati PM, Lombardi F. Efficacy of acupuncture preventing atrial fibrillation recurrences after electrical cardioversion. J Cardiovasc Electrophysiol. 2011;22:241–7. 10.1111/j.1540-8167.2010.01878.x 20807278

[12] Yang L, Yang J, Wang Q, Chen M, Lu Z, Chen S, et al Cardioprotective effects of electroacupuncture pretreatment on patients undergoing heart valve replacement surgery: a randomized controlled trial. Ann Thorac Surg. 2010;89:781–6. 10.1016/j.athoracsur.2009.12.003 20172127

[13] Kristen AV, Schuhmacher B, Strych K, Lossnitzer D, Friederich HC, Hilbel T, et al Acupuncture improves exercise tolerance of patients with heart failure: a placebo-controlled pilot study. Heart. 2010;96:1396–400. 10.1136/hrt.2009.187930 20554511

[14] Mehta PK, Polk DM, Zhang X, Li N, Painovich J, Kothawade K, et al A randomized controlled trial of acupuncture in stable ischemic heart disease patients. Int J Cardiol. 2014;176:367–374. 10.1016/j.ijcard.2014.07.011 25103909 PMC4160354

[15] Kim DD, Pica AM, Duran RG, Durán WN. Acupuncture reduces experimental renovascular hypertension through mechanisms involving nitric oxide synthases. Microcirculation. 2006;13:577–85. 10.1080/10739680600885210 16990216 PMC1618823

[16] Longhurst J. Acupuncture's cardiovascular actions: A mechanistic perspective. Med Acupunct. 2013;25:101–13. 10.1089/acu.2013.0960 24761168 PMC3616410

[17] Park JM, Shin AS, Park SU, Sohn IS, Jung WS, Moon SK. The acute effect of acupuncture on endothelial dysfunction in patients with hypertension: A pilot, randomized, double-blind, placebo-controlled crossover trial. J Altern Complement Med. 2010;16:883–8. 10.1089/acm.2009.0427 20673141

[18] Lee S, Kim W, Park JM, Jang HH, Lee SM, Woo JS, et al Effects of electroacupuncture on endothelial function and circulating endothelial progenitor cells in patients with cerebral infarction. Clin Exp Pharmcol Physiol. 2015;42:822–7. 10.1111/1440-1681.12413 25932899

[19] Kim W, Jeong MH, Cho SH, Yun JH, Chae HJ, Ahn YK, et al Effect of green tea consumption on endothelial function and circulating endothelial progenitor cells in chronic smokers. Circ J. 2006;70:1052–7. 16864941 10.1253/circj.70.1052

[20] Choi EY, Lee H, Woo JS, Jang HH, Hwang SJ, Kim HS, et al Effect of onion peel extract on endothelial function and endothelial progenitor cells in overweight and obese individuals. Nutrition. 2015;31:1131–5. 10.1016/j.nut.2015.04.020 26233871

[21] Goldman N, Chen M, Fujita T, Xu Q, Peng W, Liu W, et al Adenosine A1 receptors mediate local anti-nociceptive effects of acupuncture. Nat Neurosci. 2010;13:883–8. 10.1038/nn.2562 20512135 PMC3467968

[22] Lee S, Lee MS, Choi JY, Lee SW, Jeong SY, Ernst E. Acupuncture and heart rate variability: A systematic review. Auton Neurosci. 2010;155:5–13. 10.1016/j.autneu.2010.02.003 20304708

[23] Inoue M, Sato EF, Nishikawa M, Park AM, Kira Y, Imada I, et al Cross talk of nitric oxide, oxygen radicals, and superoxide dismutase regulates the energy metabolism and cell death and determines the fates of aerobic life. Antioxid Redox Signal. 2003;5:475–84. 10.1089/152308603768295221 13678536

[24] Xue X, You Y, Tao J, Ye X, Huang J, Yang S, et al Electro-acupuncture at points of Zusanli and Quchi exerts anti-apoptotic effect through the modulation of PI3K/Akt signaling pathway. Neurosci Lett. 2014;558:14–9 10.1016/j.neulet.2013.10.029 24157854

[25] Kim JH, Choi KH, Jang YJ, Bae SS, Shin BC, Choi BT, et al Electroacupuncture acutely improves cerebral blood flow and attenuates moderate ischemic injury via an endothelial mechanism in mice. PLoS One. 2013;8:e56746. 23418594 10.1371/journal.pone.0056736 PMC3572074

[26] Oldenburg O, Qin Q, Krieg T, Yang XM, Philipp S, Critz SD, et al Bradykinin induces mitochondrial ROS generation via NO, cGMP, PKG, and mitoKATP channel opening and leads to cardioprotection. Am J Physiol Heart Circ Physiol. 2004;286:H468–76. 10.1152/ajpheart.00360.2003 12958031

[27] O'Rourke B. Evidence for mitochondrial k+ channels and their role in cardioprotection. Circ Res. 2004;94:420–32. 10.1161/01.RES.0000117583.66950.43 15001541 PMC2712129

[28] Xiao CY, Hara A, Yuhki K, Fujino T, Ma H, Okada Y, et al Roles of prostaglandin i(2) and thromboxane a(2) in cardiac ischemia-reperfusion injury: A study using mice lacking their respective receptors. Circulation. 2001;104:2210–5. 11684633 10.1161/hc4301.098058

[29] Shinmura K, Xuan YT, Tang XL, Kodani E, Han H, Zhu Y, et al Inducible nitric oxide synthase modulates cyclooxygenase-2 activity in the heart of conscious rabbits during the late phase of ischemic preconditioning. Circ Res. 2002;90:602–8. 11909825 10.1161/01.res.0000012202.52809.40

[30] Chun KS, Cha HH, Shin JW, Na HK, Park KK, Chung WY, et al Nitric oxide induces expression of cyclooxygenase-2 in mouse skin through activation of NF-kappaB. Carcinogenesis. 2004;25:445–54. 10.1093/carcin/bgh021 14633657

[31] Lau WK, Chan WK, Zhang JL, Yung KK, Zhang HQ. Electroacupuncture inhibits cyclooxygenase-2 up-regulation in rat spinal cord after spinal nerve ligation. Neuroscience. 2008;155:463–8. 10.1016/j.neuroscience.2008.06.016 18606213

