# Supplementary figures and images for: Electroacupuncture prevents endothelial dysfunction induced by ischemia-reperfusion injury via a cyclooxygenase-2-dependent mechanism: A randomized controlled crossover trial
Source: PLoS One. 2017 Jun 7;12(6):e0178838. doi: 10.1371/journal.pone.0178838 (PMC5462401; doi:10.1371/journal.pone.0178838)

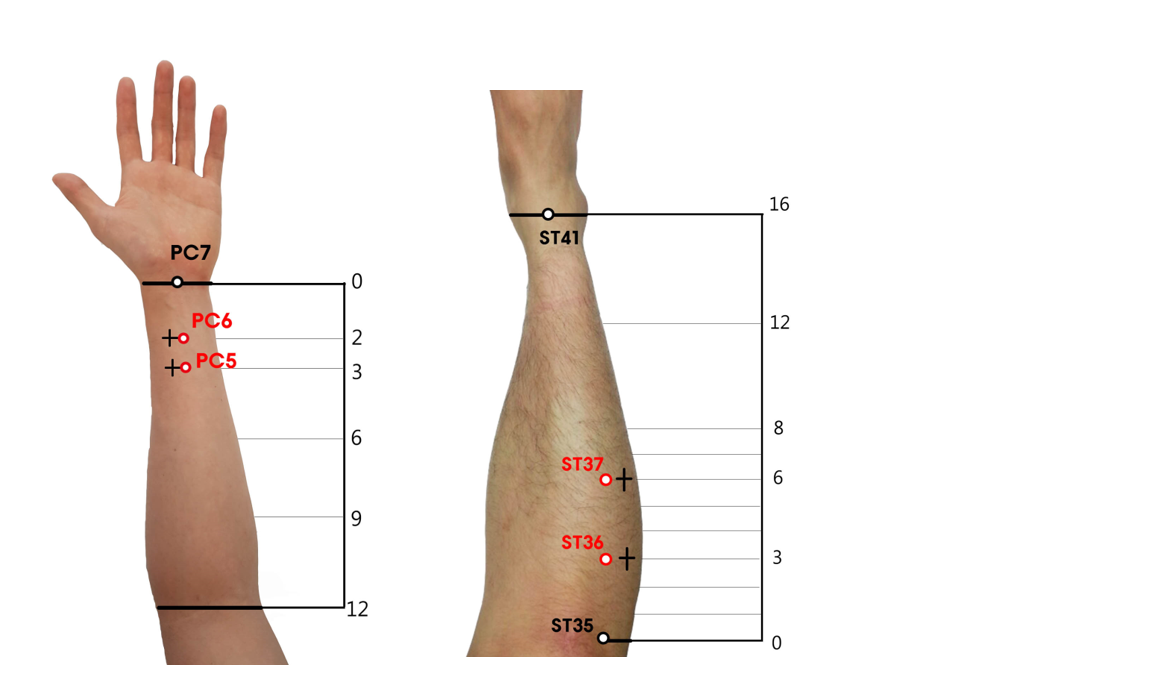

Supplement: S1 Fig — PC5 & PC6, ST36 & ST37, and sham electroacupuncture points. PC: pericardium, ST: stomach. (TIF) [file pone.0178838.s001.tif]

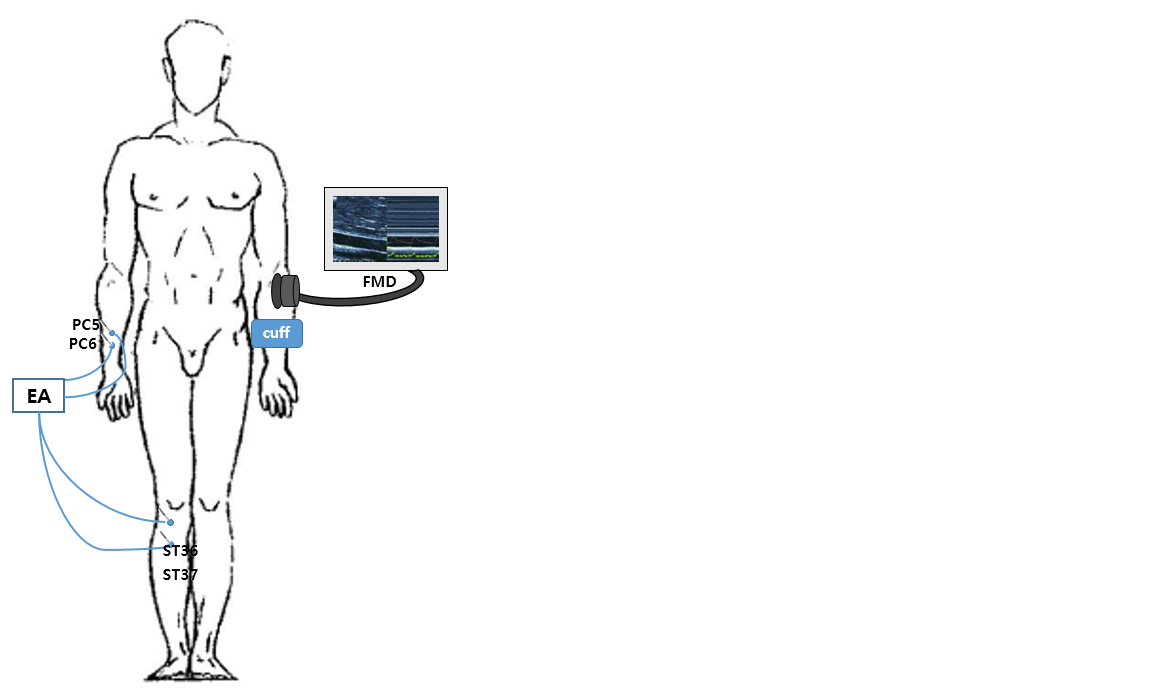

Supplement: S2 Fig — Diagram showing how electroacupuncure was conducted and how flow-mediated dilatation was measured on subjects. FMD: flow-mediated dilatation, IR: ischemia-reperfusion. (TIF) [file pone.0178838.s002.tif]
