# Supplementary material for: Electroacupuncture prevents endothelial dysfunction induced by ischemia-reperfusion injury via a cyclooxygenase-2-dependent mechanism: A randomized controlled crossover trial
Source: PLoS One. 2017 Jun 7;12(6):e0178838. doi: 10.1371/journal.pone.0178838 (PMC5462401; doi:10.1371/journal.pone.0178838)
Supplement: S2 File — Original Korean version of trial protocol. (DOC) [file pone.0178838.s004.doc]

**연 구 계 획 서**

| **허혈-재관류 손상에 의한 혈관내피세포 기능부전에 대한**  **침치료의 효과**  **Protocol No. AURORA v1.2** |
| --- |

**2014년 03월 28일**

경희대학교 의과대학 순환기내과 부교수 김 원

경희대학교 한의과대학 교수 이상훈

**연구 계획서 목차**

1. 연구의 명칭 및 기관
2. 연구의 실시기관명 및 주소
3. 연구비 지원기관명,
4. 연구의 책임자, 담당자, 공동연구자
5. 연구의 목적 및 배경
6. 대상 질환
7. 대상 연구 기간
8. 연구 방법
9. 시험대상자에 대한 안정성의 배려
10. 모니터링 계획
11. 연구기간 일정표
12. 참고문헌

- **Protocol No. : AURORA**
- **Version No. : 1.2**

1. **연구의 명칭 및 기관**

- 명칭

국문 ) 허혈-재관류 손상에 의한 혈관내피세포 기능 부전에 대한 침치료의 효과

- 기관

경희대 의대 순환기내과

경희대 한의대 침구과

- 연구디자인

본 연구는 단일기관, 무작위배정, 위약 대조, 교차 시험 연구이다.

1. **연구의 실시 기관명 및 주소**
   - 기관명 : 경희의대 순환기내과, 경희대 한의대 침구과
   - 주소 : 서울시 동대문구 회기동 1번지, 대표전화 : 02-958-8169
2. **연구비 지원 기관명**
   - 보건복지부 한의선도과제 한양방협력기술개발사업.
3. 연구의 책임자, 담당자, 공동연구자

| **책임연구자** | 김원 | 순환기내과 | 부교수 | 순환기내과 |
| --- | --- | --- | --- | --- |
|  | Tel : 02-958-8169 E-mail : mylovekw@hanmail.net | | | |
| **공동연구자** | 이상훈 | 경희 한의대 | 교수 | 침구과 |
|  | 김권삼 | 순환기내과 | 교수 | 순환기내과 |
|  | 김우식 | 순환기내과 | 부교수 | 순환기내과 |
|  | 김진배 | 순환기내과 | 조교수 | 순환기내과 |
|  | 김현수 | 순환기내과 | 전임의 | 순환기내과 |
|  | 우종신 | 순환기내과 | 전임의 | 순환기내과 |

**5.** **연구의 목적 및 배경**

- **연구목적**

침치료가 허혈-재관류 손상시에 혈관내피세포 의존성 혈관 손상에 미치는 효과와 기전을 알아본다.

- **연구배경** **및 목적**

혈관내피세포의 기능 이상은 다양한 심혈관 질환의 발생, 진행, 그리고 예후와 관련이 있다. 그래서 전통적인 심혈관계 위험인자인 당뇨, 고혈압 및 고지혈증의 조절뿐 아니라 혈관내피기능의 이상은 조절되어야 할 문제로 생각되고 있다.

허혈 재관류에 의해 야기되는 병태생리학적인 기전으로는 혈관의 톤(vasomotor), 혈전, 염증등이 각광을 받고 있으며 이에 내막의 역할이 강조되고 있다. 내막 세포(Endothelial cells)는 심근(myocyte)보다 허혈 재관류 손상에 민감하며 허혈시에 내막이 특정자극에 반응성이 떨어지는 것 (내막 기능 장애 : “endothelial dysfunction”)은 시간적으로 허혈 재관류 손상에 의한 조직 손상보다 앞서서 온다고 알려져 있다. 여러 연구에서 허혈 이전에 짧은 반복적인 preconditioning (ie. ischemic preconditioning)이나 약물적인 전처치(chemical preconditioning)가 이러한 허혈 재관류 손상에 대한 내막의 반응도를 호전시킨다는 증거가 제시되고 있다.

전침 치료는 인체의 경혈점에 침을 자입한 후 전기자극을 연결하는 치료법으로서 통증, 고혈압, 심장질환의 치료에 많이 활용되고 있으며, 실험 및 임상연구도 비교적 많이 시행되어 있다. 최근 침 치료(족삼리와 내관에 자침)가 고혈압 환자에서 혈관내피기능을 반영하는 FMD를 호전시킨다고 보고된 바 있으며, 이는 NO의 생성과 활성의 증가에 기인한다고 한다. 또한 침 자극은 심혈관계에도 다양한 영향을 미친다고 보고되고 있으며, 특히 족삼리 자침이 혈관벽의 긴장도를 유지시키는데 핵심적인 역할을 하는 혈관내피세포유래 NO의 생성과 활성을 증가시킴으로써 심혈관계에 긍정적인 효능을 미친다고 보고되었다. 그러나, 사람에서 유발된 허혈-재관류 손상에 따른 내피세포기능장애에 대한 침 치료의 효과가 있는지는 아직 연구가 이루어지지 않았다.

그러므로 본 연구자는 사람에서 허혈 재관류 손상시에 야기되는 내막 장애 모델을 통해 전침치료가 허혈 재관류 손상시에 endothelium 의존성 혈관 확장에 미치는 효과를 얼마나 극복하는 지를 알아보고자 한다.

**6. 대상환자 : 선정 및 제외기준**

건강한 성인 남녀를 대상으로 한다.

1. 만 25세 이상 40세 미만 건강한 남녀 (비흡연자)
2. 서면 동의서에 서명한 자
3. 제외기준

1. 고혈압 (>140/90 mmHg), 당뇨병 등 심혈관 질환자.

2. 간기능 이상자 (bilirubin level >2 mg/dl)

3. 신장 기능 이상자 (Cr > 정상상한치의 2배)

4. 갑상선 기능 이상자

5. 뇌혈관계 질환자

6. 임산부

7. BMI > 25 kg/m2

**7. 대상 연구 기간**

전체 약 8개월간 시행 예정. 시험대상자 등록 시작은 2014년 5월부터 시작하여, 2014년 12월 마지막 시험대상자 실험을 종료하는 것을 목표로 한다.

**8. 연구 방법**

- - **연구 방법 개요 (Figure 1,2)**

1) 정상 시험대상자를 선별하여, 기본시점에서 혈압, 심전도, 혈관내피 기능 검사 (FMD), 채혈을 한다. 인간의 상완을 이용한 허혈-재관류 손상은 확립된 모델로서 다음과 같이 만든다. 우측 상완을 혈압계의 cuff를 200mmHg 이상 감아 15분동안 혈류를 완전히 차단하여 허혈상태를 만든다. 그 후, 15분간 풀어 재관류 상태를 만들어 허혈 후 재관류 손상을 유발한다. 손상정도는 허혈-재관류 손상 전,후로 혈관확장반응(flow-mediated dilation; FMD)을 측정하여 수치를 하여 정한다.

2) 무작위 배정을 통해 acupuncture 군과 placebo군으로 대상자를 나누어 acupuncture 군은 재관류 5분전부터 20분간 전침 치료를 수행하고, placebo군은 20분간 placebo치료를 시행한다. 침치료 직후 5분 동안 안정 후, 혈압, 심전도, 혈관내피 기능 검사 (FMD)를 시행한다.

3) 1주일 후 이전에 placebo군이었던 환자는 acupuncture군으로 이전에 acupuncture군이었던 환자는 placebo군으로 배정하여 위의 검사와 침치료를 반복한다. 침치료 시행한 군이 시행하지 않은 군과 비교하여 혈관내피 기능이 손상되지 않는지를 확인한다.(Fig 1)

4) 8명의 건강한 실험 대상자를 선발하여 5mg glibenclamide (Euglucon, Roche Pharma)를 FMD 측정 3시간 전에 투여한 후 프로토콜 1 방식대로 재실험한다. 상기 glibenclamide의 용량은 KATP 채널을 완전히 억제할 수 있는 것으로 밝혀져 있으며, 혈당 수치를 떨어뜨릴 수 있으므로 모든 피험자는 약물 투여 후 혈당 80~120 mg/dL 이 유지되도록 10% dextrose 를 투여하며 모니터링 하도록 한다. 허혈-재관류 기간 동안 위 방식대로 20분간 침치료 한다.

5) 8명의 건강한 실험 대상자를 선발하여 COX-2 억제제인 celecoxib 을 500mg씩 하루에 2회 총 5일동안 복용하고 마지막 투여일 오전에 허혈-재관류 기간 동안 위 방식대로 20분간 침치료 한다.


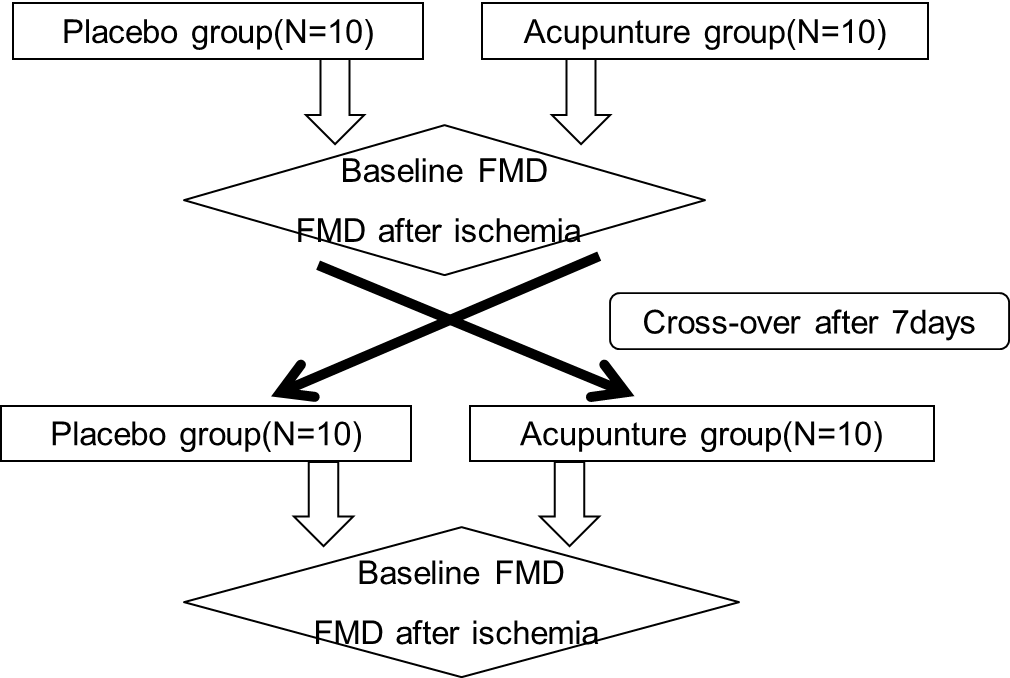


**Fig 1. 연구 디자인**

**
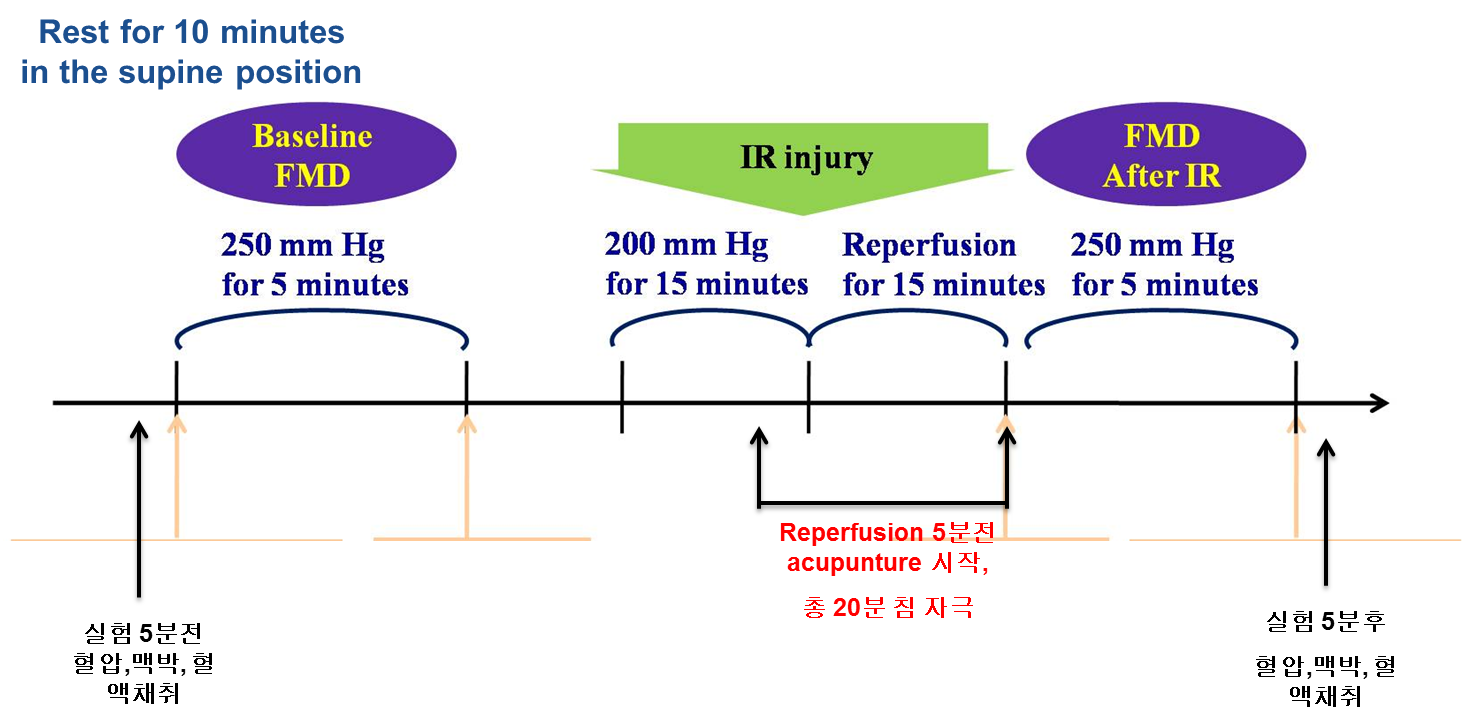
**

**Fig 2. 연구 프로토콜**

**1) 침 시술 방법**

1. 동의서 취득 후 검사시간을 예약하고, 해당 예약시간에 시험대상자는 심장초음파실에서 baseline 검사로서 초음파를 이용한 혈관내피기능검사(FMD)를 시행한다.

2. 시술은 침상에 누운 안정된 상태에서 실시한다. 검사 당일 환자가 심장초음파방으로 내려오면 5분 안정 혈압, 심전도를 측정한다. (심전도에 측정한 혈압을 기록) 5분 안정 후 FMD, CFR 을 측정한다. 그 후, 배정된 군에 따라 20분간 전침 치료 또는 placebo 침치료를 한다. 치료 후 5분간 휴식하고, F/U 채혈, 혈압, 심전도를 측정하고, 그 후 FMD, CFR을 재측정 한다.

3. 사용경혈은 족삼리(足三里)와 상거허(上巨虛), 내관(內關)과 간사(間使)로서, 1회용 멸균 호침(동방침구사, 한국, 0.25x40mm)을 2±0.5cm 깊이로 자입하여 득기감을 유도하고, 이후 별도의 추가적 수기 자극은 실시하지 않는다.

4. 저주파자극기(ES-160, ITO, Japan)의 채널을 자침한 족삼리(足三里)와 상거허(上巨虛), 내관(內關)과 간사(間使)에 연결한다.

5. 2Hz 빈도로 20분간 단상파 펄스형의 전침 자극을 준다. 강도는 저강도부터 서서히 증가시켜 환자가 자극을 느끼고 근육의 가벼운 수축이 일어나는 정도로 하며, 통증 역치 이하의 불편감을 느끼지 않을 정도의 강도로 한다.

6. 이상반응 발생 시 1차적으로 안정 및 회복 시까지 근접, 관찰하고, 필요 시 추가적인 조치를 취한다.

7. 가침 자극은 경혈이 아닌 인접부위를 침으로 자입하여 전침군과 똑같이 2Hz 빈도로 20분간 단상파 펄스형의 전침 자극을 준다.

**2) 혈관내피세포 기능 (Flow-mediated dilatation; FMD) 평가**

내피세포 기능장애의 평가는 비침습적인 방법인 상완동맥 내피세포 의존성 혈관확장반응 측정을 이용한다.. 고해상도 초음파 기계(SONOS 5500, Hewlett Packard, U.S.A)를 이용하여 B-mode 하에서 7.5 MHz 탐촉자(Hewlett Packard, U.S.A.)로 측정한다.

기저치 상완동맥 직경을 측정 후 좌측 전완 근위부에 감겨져 있는 혈압계의 압력을 250 mmHg까지 올려 상완동맥의 혈류를 차단하고, 5분 후 혈압계의 압력을 급속히 0 mmHg로 내려 상완동맥 내에 과혈류 상태를 유발한다. 혈관 직경의 측정은 앞쪽 면의 내막(intima)과 중막(media)이 만나는 접촉면(interface)부터 반대쪽 면의 내막까지로 하고, 직경 측정시의 편차를 줄이기 위하여 가능하면 기준점을 동맥분지 등이 있는 곳에 설정하고, 측정 시기는 심장 이완기 끝부분, 즉 심전도에서 R파 기시부 직전의 직경 값을 측정한다.

**3) 목표 대상자의 수 및 무작위 배정 방법: 총 20명**

목표 대상자 산출의 근거: 과거 본 연구진이 건강한 지원자를 대상으로 시행하였던 연구 결과를 살펴보면 허혈/재관류 손상 시행 전 FMD 는 12.0 ± 6.2 이고, 허혈/재관류 손상 시행 후에는 4.6 ± 3.6 이었다. ^26^ 당시 시험약제로 사용하였던 exenatide 의 경우 허혈/재관류 손상을 100% 막았으나 (허혈/재관류 손상 전 FMD =15.0 ± 7.1, 허혈/재관류 손상 후 FMD = 15.0 ± 5.9), 침 치료의 허혈/재관류 손상의 예방 효과에 대한 기존 연구는 없는 상태이다. 통상 허혈/재관류 손상에 관한 연구에서 30% 효과 ^27^ 를 기대하고 진행하는 것에 비추었을 때, 본 연구자들도 침 치료가 허혈/재관류 손상 후 FMD 를 30% 호전시킨다고 가정하며, 1종 오류를 5%, power 를 80% 로 가정할 때 목표 대상자 수는 각 군 17명으로 총 34명이다. 15% 탈락율을 고려할 때 총 40명의 환자가 필요하며, 본 연구는 crossover trial로 20명을 목표 대상자로 정하였다.

무작위배정방법은 고정 무작위배정 방법으로서 퍼뮤터블 블록 무작위배정 법을 사용한다.

**4) 관찰항목, 관찰검사 방법 및 임상검사항목**

- - - - 환자기본정보는 연구번호, 이름, 성별, 나이, 진단명, 과거력, 흡연력, 가족력, 키(cm), 체중(kg) 정보를 수집한다.
      - 환자에서 혈압, 심전도, 혈관내피 기능 검사 (FMD)를 측정한다.
- 3군의 혈소판 기능 검사를 분석하여 omeprazole의 투여가 clopidogrel의 혈소판 억제를 방해하는지 확인한다. 또한 omeprazole의 아침와 저녁의 용법의 차이가 clopidogrel의 혈소판 기능 억제에 미치는 영향에 대해서도 확인한다.
  - - - 시험대상자의 개인신상정보 보안 및 자료 보안: 연구결과에 개인신상정보는 포함되지 않으며 이를 위하여 연구결과에 코드번호를 부여하여 관리함으로써 연구결과의 출판 및 연구결과가 공개 될 때에도 개인신상정보는 포함되지 않도록 할 것이다. 또한, 책임연구자는 코드 화 되기 전 수집된 개인정보들이 누출되지 않도록 주의 감독에 대한 책임을 진다.

**5) 통계분석**

- - - - 자료분석은 SPSS (version 17.0, Chicago, Illinois, USA)을 이용한다. 통계적 유의수준은0.05로 하고, 자료는 평균±표준편차로 표시한다. 정규분포를 보이는 연속변수는 Student *t* test 또는 Paired t-test를 이용하고, 비모수적 검증은 Mann-Whitney test 또는 Wilcoxson signed rank test 등을 이용한다. 범주형 자료는 Chi-square test를 이용한다.

허혈/재관류 손상 시행 전과 후의 FMD 값의 변화는 two way ANOVA 방법을 사용한

다.

**6) 연구대상자 모집 방법**

모집 공고를 통해 정상인을 모집할 예정이다. 모집 공고는 경희대병원 및 경희대학교에 게시할 예정이다.

**9. 시험대상자에 대한 안전성의 배려**

본 연구는 검진, 병력청취, 혈관내피세포 기능검사 등을 시행하고 추가로 시행되는 침습적인 시술이나 위험한 투약은 없어 안전성에 큰 문제는 없을 것으로 판단됩니다.

15분간 혈압계를 이용하여 압박을 시행할 때 압박에 대한 통증 및 청색증이 있을 수 있으며, 의료진이 근접 관찰 할 예정이며, 청색증이 심하다고 판단될 때에는 혈압계로 인한 압박을 중단할 수 있습니다.

Acupuncture 시술은 현재 한의계에서 사용되는 일반적인 시술방법으로서 안전성은 확보되어 있습니다. 알려진 침술의 부작용은 시술 부위의 국소 통증, 출혈, 자반, 홍반, 일시적인 어지러움증, 오심 등이 있을 수 있으나, 대체로 수시간에서 수일 내에 자연 회복되며, 드물게 내부장기의 손상이 있을 수 있으나, 본 연구에서는 체간이 아닌 팔다리의 경혈만을 사용하므로, 이러한 장기에 대한 손상 가능성은 없습니다.

국소 통증, 출혈, 자반, 홍반, 일시적인 어지러움, 오심 등의 부작용 발생시에는 1차적으로 안정 및 회복시까지 근접, 관찰할 예정이며, 추가적인 검사 및 치료를 요하는 침술과 관련된 부작용이 객관적으로 입증되면 자체 연구비에서 검사비 및 치료비를 보상할 계획입니다

**10. 모니터링 계획**

시험대상자의 권리와 복지 보호, 임상시험관련 자료가 근거 문서와 대조하여 정확하고 완전하며, 검증 가능한지 여부 확인, 임상시험계획서 준수 여부 및 관련법규를 준수하여 임상시험을 진행하였는지를 확인하기 위해 시험책임자는 임상시험심사위원회에 점검(audit) 및 모니터링(monitoring)을 신청하거나, 임상시험심사위원회 점검 시 적극 협조한다

**11.** 연구시행 일정표

| 개월 | 1 | 2 | 3 | 4 | 5 | 6 | 7 | 8 | 9 | 10 | 11 | 12 | 13-15 |
| --- | --- | --- | --- | --- | --- | --- | --- | --- | --- | --- | --- | --- | --- |
| 대상환자 선정 및 등록 |  |  |  |  |  |  |  |  |  |  |  |  |  |
| 내피세포 측정 |  |  |  |  |  |  |  |  |  |  |  |  |  |
| 자료정리 및 분석 |  |  |  |  |  |  |  |  |  |  |  |  |  |
| 보고서 및 논문 작성 |  |  |  |  |  |  |  |  |  |  |  |  |  |

**12. 참고문헌**

[1] Scheter M, Issachar A, Marai I, Koren-Morag N, Freinark D, Shahar Y, Shechter A, Feinberg MS. Long-term association of brachial artery flow-mediated vasodilation and cardiovascular events in middle-aged subjects with no apparent heart disease. Int J Cardiol 2009;134(1):52-8

[2] Simon A, Mijiti W, Gariepy J, Levenson J. Current possibilities for detecting high risk of cardiovascular disease. Int J Cardiol 2006;110(2):146

[3] Murohara T, Asahara T, Silver M, et al. Nitric oxide synthase modulates angiogenesis in response to tissue ischemia. J Clin Invest 1998;101:2567-78.

[5] Hill JM, Zalos G, Halcox JP, et al. *Circulating endothelial progenitor cells, vascular function, and cardiovascular risk. N Engl J Med 2003;348:593-600.*

[6] Gokce N, Keaney JF, Jr, Hunter LM, Watkins MT, Menzoian JO, Vita JA. *Risk stratification for postoperative cardiovascular events via noninvasive assessment of endothelial function: a prospective study. Circulation 2002;105:1567-72.*

[7] Widlansky ME, Gokce N, Keaney JF, Jr, Vita JA. *The clinical implications of endothelial dysfunction. J Am Coll Cardiol 2003;42:1149-60.*

[8] Cao Q, Liu J, Chen S, Han Z. Effects of electroacupuncture at neiguan on myocardial microcirculation in rabbits with acute myocardial ischemia. J Tradit Chin Med. 1998 Jun;18(2):134-9.

[9] Wu HC, Lin JG, Chu CH, Chang YH, Chang CG, Hsieh CL, Tsai AH, Ueng KC, Kuo WW, Lin JA, Liu JY, Huang CY. The effects of acupuncture on cardiac muscle cells and blood pressure in spontaneous hypertensive rats. Acupunct Electrother Res. 2004;29(1-2):83-95.

[10] Williams T, Mueller K, Cornwall MW. Effect of acupuncture-point stimulation on diastolic blood pressure in hypertensive subjects: a preliminary study. Phys Ther. 1991 Jul;71(7):523-9.

[11] Saku K, Mukaino Y, Ying H, Arakawa K. Characteristics of reactive electropermeable points on the auricles of coronary heart disease patients. Clin Cardiol. 1993 May;16(5):415-9.

[12] Tukmachi E, Jubb R, Dempsey E, Jones P. The effect of acupuncture on the symptoms of knee osteoarthritis--an open randomised controlled study. Acupunct Med. 2004 Mar;22(1):14-22.

[13] Park JM, Shin AS, Park SU, Sohn IS, Jung WS, Moon SK. The acute effect of acupuncture on endothelial dysfunction in patients with hypertension: a pilot, randomized, double-blind, placebo-controlled crossover trial. J Altern Complement Med. 2010 Aug;16(8):883-8.

[14] Kim DD, Pica AM, Durán RG, Durán WN. Acupuncture reduces experimental renovascular hypertension through mechanisms involving nitric oxide synthases. Microcirculation. 2006 Oct-Nov;13(7):577-85.

[15] Chen S, Ma SX. Nitric oxide in the gracile nucleus mediates depressor response to acupuncture (ST36). J Neurophysiol. 2003 Aug;90(2):780-5. Epub 2003 Apr 2.

[16] Li L, Yin-Xiang C, Hong X, Peng L, Da-Nian Z. Nitric oxide in vPAG mediates the depressor response to acupuncture in stress-induced hypertensive rats. Acupunct Electrother Res. 2001;26(3):165-70.

[18] Li P, Sun FY, Zhang AZ. The effect of acupuncture on blood pressure: the interrelation of sympathetic activity and endogenous opioid peptides. Acupunct Electrother Res. 1983;8(1):45-56.

[19] Mori H, Uchida S, Ohsawa H, Noguchi E, Kimura T, Nishijo K. Electro-acupuncture stimulation to a hindpaw and a hind leg produces different reflex responses in sympathoadrenal medullary function in anesthetized rats. J Auton Nerv Syst. 2000 Mar 15;79(2-3):93-8.

[20] Yao T. Acupuncture and somatic nerve stimulation: mechanism underlying effects on cardiovascular and renal activities. Scand J Rehabil Med Suppl. 1993;29:7-18.

[21] Michikami D, Kamiya A, Kawada T, Inagaki M, Shishido T, Yamamoto K, Ariumi H, Iwase S, Sugenoya J, Sunagawa K, Sugimachi M. Short-term electroacupuncture at Zusanli resets the arterial baroreflex neural arc toward lower sympathetic nerve activity. Am J Physiol Heart Circ Physiol. 2006 Jul;291(1):H318-26. Epub 2006 Feb 24.

[22] Laude K, Beauchamp P, Thuillez C, Richard V. Endothelial protective effects of preconditioning. Cardiovasc Res. 2002;55:466–473.

[23] Mankad PS, Amrani M, Rothery S, Severs NJ, Yacoub MH. Relative susceptibility of endothelium and myocardial cells to ischaemiareperfusion injury. Acta Physiol Scand. 1997;161:103–112.

[24] Tomai F, Crea F, Chiariello L, Gioffre PA. Ischemic preconditioning in humans: models, mediators, and clinical relevance. Circulation. 1999; 100:559–563.

[25] Beresewicz A, Maczewski M, Duda M. Effect of classic preconditioning and diazoxide on endothelial function and O2 _ and NO generation in thepost-ischemic guinea-pig heart. Cardiovasc Res. 2004;63:118–129.

[26] Ha SJ, Kim W, Woo JS, Kim JB, Kim SJ, Kim WS, Kim MK, Cheng XW, Kim KS. Preventive effects of exenatide on endothelial dysfunction induced by ischemia-reperfusion injury via KATP channels. Arterioscler Thromb Vasc Biol. 2012;32:474-480.

# [27] [**Piot C**](http://www.ncbi.nlm.nih.gov/pubmed?term=Piot C%5BAuthor%5D&cauthor=true&cauthor_uid=18669426), [**Croisille P**](http://www.ncbi.nlm.nih.gov/pubmed?term=Croisille P%5BAuthor%5D&cauthor=true&cauthor_uid=18669426), [**Staat P**](http://www.ncbi.nlm.nih.gov/pubmed?term=Staat P%5BAuthor%5D&cauthor=true&cauthor_uid=18669426), [**Thibault H**](http://www.ncbi.nlm.nih.gov/pubmed?term=Thibault H%5BAuthor%5D&cauthor=true&cauthor_uid=18669426), [**Rioufol G**](http://www.ncbi.nlm.nih.gov/pubmed?term=Rioufol G%5BAuthor%5D&cauthor=true&cauthor_uid=18669426), [**Mewton N**](http://www.ncbi.nlm.nih.gov/pubmed?term=Mewton N%5BAuthor%5D&cauthor=true&cauthor_uid=18669426), [**Elbelghiti R**](http://www.ncbi.nlm.nih.gov/pubmed?term=Elbelghiti R%5BAuthor%5D&cauthor=true&cauthor_uid=18669426), [**Cung TT**](http://www.ncbi.nlm.nih.gov/pubmed?term=Cung TT%5BAuthor%5D&cauthor=true&cauthor_uid=18669426), [**Bonnefoy E**](http://www.ncbi.nlm.nih.gov/pubmed?term=Bonnefoy E%5BAuthor%5D&cauthor=true&cauthor_uid=18669426), [**Angoulvant D**](http://www.ncbi.nlm.nih.gov/pubmed?term=Angoulvant D%5BAuthor%5D&cauthor=true&cauthor_uid=18669426), [**Macia C**](http://www.ncbi.nlm.nih.gov/pubmed?term=Macia C%5BAuthor%5D&cauthor=true&cauthor_uid=18669426), [**Raczka F**](http://www.ncbi.nlm.nih.gov/pubmed?term=Raczka F%5BAuthor%5D&cauthor=true&cauthor_uid=18669426), [**Sportouch C**](http://www.ncbi.nlm.nih.gov/pubmed?term=Sportouch C%5BAuthor%5D&cauthor=true&cauthor_uid=18669426), [**Gahide G**](http://www.ncbi.nlm.nih.gov/pubmed?term=Gahide G%5BAuthor%5D&cauthor=true&cauthor_uid=18669426), [**Finet G**](http://www.ncbi.nlm.nih.gov/pubmed?term=Finet G%5BAuthor%5D&cauthor=true&cauthor_uid=18669426), [**André-Fouët X**](http://www.ncbi.nlm.nih.gov/pubmed?term=André-Fouët X%5BAuthor%5D&cauthor=true&cauthor_uid=18669426), [**Revel D**](http://www.ncbi.nlm.nih.gov/pubmed?term=Revel D%5BAuthor%5D&cauthor=true&cauthor_uid=18669426), [**Kirkorian G**](http://www.ncbi.nlm.nih.gov/pubmed?term=Kirkorian G%5BAuthor%5D&cauthor=true&cauthor_uid=18669426), [**Monassier JP**](http://www.ncbi.nlm.nih.gov/pubmed?term=Monassier JP%5BAuthor%5D&cauthor=true&cauthor_uid=18669426), [**Derumeaux G**](http://www.ncbi.nlm.nih.gov/pubmed?term=Derumeaux G%5BAuthor%5D&cauthor=true&cauthor_uid=18669426), [**Ovize M**](http://www.ncbi.nlm.nih.gov/pubmed?term=Ovize M%5BAuthor%5D&cauthor=true&cauthor_uid=18669426). Effect of cyclosporine on reperfusion injury in acute myocardial infarction. [**N Engl J Med.**](http://www.ncbi.nlm.nih.gov/pubmed?term=359%5Bvolume%5D+AND+473%5Bpage%5D+AND+2008%5Bpdat%5D&cmd=detailssearch)**2008;359:473-81.**
