# Supplementary material for: Electroacupuncture prevents endothelial dysfunction induced by ischemia-reperfusion injury via a cyclooxygenase-2-dependent mechanism: A randomized controlled crossover trial
Source: PLoS One. 2017 Jun 7;12(6):e0178838. doi: 10.1371/journal.pone.0178838 (PMC5462401; doi:10.1371/journal.pone.0178838)
Supplement: S3 File — Translated version of original trial protocol. (DOCX) [file pone.0178838.s005.docx]

**Research Protocol**

| **Effect of Acupuncture to endothelial dysfunction induced by ischemia-Reperfusion injury (AURORA Study)**  **Protocol No. AURORA v1.2** |
| --- |

**2014. 03. 28**

**Kyunghee University Medicine College Department of Cardiology Associate Professor Weon Kim**

**Kyunghee University Oriental Medicine College Department of Acupuncture and Moxibustion**

**Professor Sanghoon Lee**

**Table of contents**

1. Research title and participating organ
2. Name and address of participating organ
3. Funding institution
4. Chief, manager and co-researcher
5. Purpose and background
6. Targeted subjects
7. Research period
8. Research Methods
9. Safety of Subjects
10. Monitoring plan
11. Research timetable
12. Reference

- Protocol No. : AURORA
- Version No. : 1.2

1. **Research title and participating departments**

- Title

Effect of Acupuncture to endothelial dysfunction induced by ischemia-reperfusion injury (AURORA Study)

- Departments

Kyung Hee University, Division of Cardiology, Department of Internal Medicine

Kyung Hee University, College of Korean Medicine, Department of Acupuncture and Moxibustion

- Design

Single center, prospective, randomized, placebo-controlled, cross-over trial

**2. Name and address of participating departments**

- 1. Name :

Kyung Hee University, Division of Cardiology, Department of Internal Medicine

Kyung Hee University, College of Korean Medicine, Department of Acupuncture and Moxibustion

- 1. Address : 1st, Hoegi-dong, Dongdaemun-gu, Seoul, Republic of Korea Seoul

Phone number 02-958-8169

**3. Name of funding organization**

- 1. Traditional Korean Medicine R&D Project Ministry of Health & Welfare, Korea

| **Principal**  **Investigator** | Weon Kim | Kyung Hee University Division of Cardiology | Associate professor | Department of  Internal Medicine |
| --- | --- | --- | --- | --- |
|  | **T**el : 02-958-8169 E-mail : mylovekw@hanmail.net | | | |
| **Co-researcher** | Sanghoon Lee | Kyunghee University College of Korean Medicine | Professor | Department of Acupuncture and Moxibustion |
|  | Kwon Sam Kim | Kyunghee University Division of Cardiology | Professor | Department of  Internal Medicine |
|  | Woo-Shik Kim | Kyunghee University Division of Cardiology | Associate professor | Department of  Internal Medicine |
|  | Jin-Bae Kim | Kyunghee University Division of Cardiology | Assistant professor | Department of  Internal Medicine |
|  | Hyun Soo Kim | Kyunghee University Division of Cardiology | Fellow | Department of  Internal Medicine |
|  | Jong Shin Woo | Kyunghee University Division of Cardiology | Fellow | Department of  Internal Medicine |

**4. Principal investigator and coresearchers**

**5. Purpose and background**

● Purpose

This study aims to explore the potential effect and mechanisms of electroacupuncture (EA) in ischemia reperfusion (IR)-induced endothelial dysfunction in humans.

● Background

Chronic dysfunction of endothelial cells leads to atherosclerosis and also acts as an important independent predictor of negative prognosis for coronary artery diseases. The dysfunction of endothelial cells is considered a risk factor for circulatory diseases that needs to be controlled, as well as diabetes, hypertension and hyperlipidemia.

As vasomotor, thrombosis and inflammation are important in IR injury mechanism, endothelial function is in the spotlight. Endothelial cells are more sensitive to IR injury than cardiomyocytes. Under ischemic circumstances, endothelial dysfunction occurs earlier then tissue injury due to IR damage. In many studies, repetitive short-term preconditioning (ie. ischemic preconditioning) or chemical preconditioning before ischemia have been reported to improve the endothelial function after IR injury.

EA is commonly used to alleviate pain but it has also been widely explored in the treatment of hypertension and many cardiovascular diseases. A preliminary study in patients with hypertension suggested that acupuncture improved flow-mediated dilation (FMD), and that it might result from enhancement of generation and bio-activity of nitric oxide (NO). Acupuncture has been reported to have variable effects on cardiovascular system and especially needling on ST36 have been known to have beneficial effects on the cardiovascular system by increasing production of endothelial NO. Since then no other clinical study has investigated how acupuncture might be able to protect against IR-induced endothelial dysfunction in a human model.

Therefore we designed two protocols to test whether acupuncture can prevent impairment in IR-induced endothelial dysfunction in a human forearm model of IR-induced endothelial dysfunction.

**6. Participants : Inclusion and exclusion criteria**

Healthy adults, both men and women (non-smokers)

① Above 25 and below 40 years

② Those who provide written consent

③ Exclusion criteria

1. Those who suffer from chronic diseases such as Hypertension (>140/90 mmHg) or Diabetes

2. Abnormal liver function (bilirubin level >2 mg/dl)

3. Abnormal kidney function (Cr > twofold of upper normal limit)

4. Thyroid related disease

5. Cerebrovascular disease

6. Pregnant women

7. Body mass index >25 kg/m2

**7. Research period**

Approximately 8 months. Enrollment of subjects starts from May, 2014. Final study on the last subjects is estimated to end on December, 2014.

**8. Research methods**

- Overview (Figure 1,2)

1) Endothelium-dependent, FMD of the brachial artery will be measured in healthy volunteers before and after IR (15 minutes of ischemia at the level of the proximal upper arm with more than 200mmHg cuff pressure followed by 15 minutes of reperfusion).

2) 20 healthy nonsmoking volunteers will be randomly assigned to acupuncture or control. In the acupuncture group, acupuncture will be performed from 5 minute before reperfusion till the end time of reperfusion for 20 minutes. In the control group, subjects will receive placebo treatment for 20 minutes. With 5 minutes rest after acupuncture, blood pressure, ECG, FMD will be performed.

3) One week later, each group will be crossed-over to the other group. We will test whether the endothelial function is less impaired in the acupuncture group, compared to the control group.(Fig 1)

4) 8 healthy volunteers are administered 5 mg of glibenclamide (Euglucon, Roche Pharma) 3 hour before FMD measurement. This dosage has previously been shown to be able to completely inhibit forearm KATP channels. With the glibenclamide administration, a 10% dextrose infusion is started and titrated to maintain blood sugar levels between 80 and 120 mg/dL throughout the study period. 3 hours after glibenclamide administration, the subjects undergo FMD measurement before and after ischemia reperfusion injury. During ischemia reperfusion period, active acupuncture treatment is performed for 20 minutes like above method.

5) 8 healthy volunteers are administered celecoxib, a selective COX-2 inhibitor, 200 mg twice daily for 5 days. Last dose of celecoxib is administered at morning. Volunteers undergo FMD measurements before and after ischemia reperfusion injury in that morning of last dose of celecoxib. During ischemia reperfusion period, active acupuncture treatment is performed for 20 minutes like above method


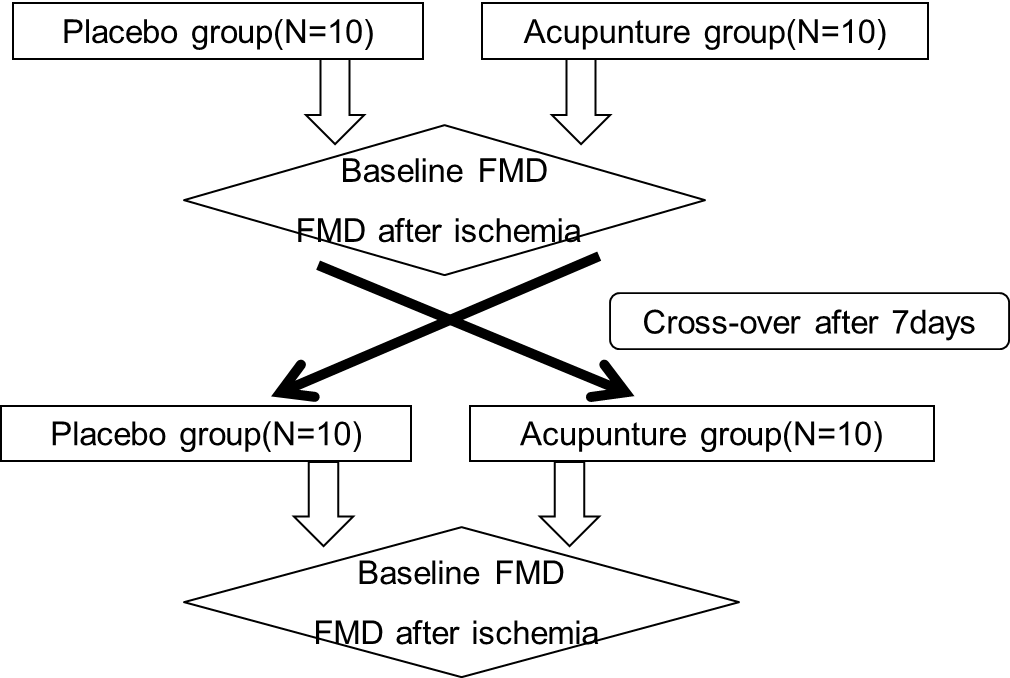


Fig 1. Research design

**
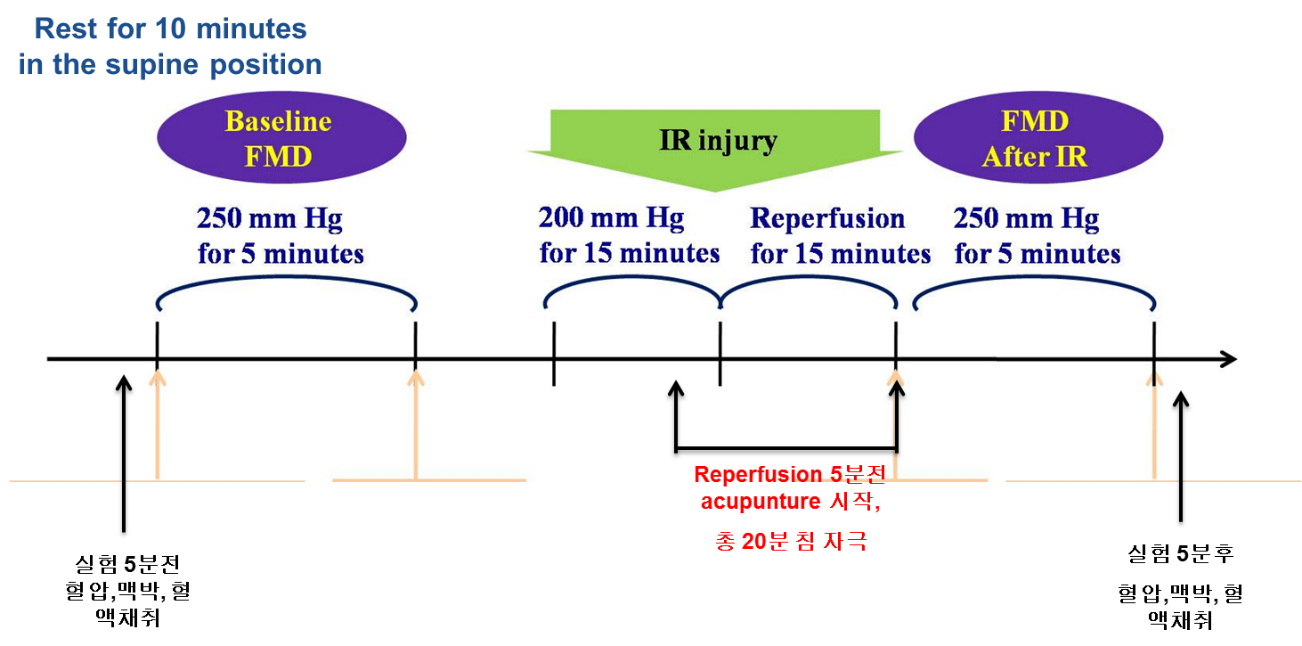
**

Fig 2. Research protocol

**1) Intervention (Acupuncture) method**

1. After giving written consent, subjects will be asked to visit the examination room for baseline tests.

2. Subjects will be asked to rest in a supine position prior to each study protocol. First, resting blood pressure and ECG will be measured. After 5 minutes of rest, FMD and CFR will be measured. Then, intervention will be applied according to the allocated group. After 5 minutes of rest, blood pressure, ECG, FMD, CFR will be measured.

3. For EA treatment, acupuncture meridian point Pericardium5 (PC5), Pericardium6 (PC6), Stomach36 (ST36), and Stomach37 (ST37) will be chosen. Disposable, sterile needles (0.25x40mm) will be inserted unilaterally to a depth of 2±0.5cm with de-qi sensation.

4. Low frequency electrical stimulator (ES-160, ITO, Japan) will be used. Needles will be connected to the pole between the two adjacent points (PC5-PC6, ST36-ST37).

5. Electrical stimulation will be applied with a 2 Hz continuous wave current and an intensity for light muscle contraction for 20 minutes.

6. Close observation will be performed to check for adverse events. If necessary, examiners will have follow up measurements.

7. For control treatment, same EA protocol on nearby non-acupoints will be applied.

**2) Measurement of Flow-mediated dilatation (FMD)**

Measurement of endothelial dysfunction uses evaluating BA endothelial cell-mediated vasodilation response method, which is non-invasive. FMD will be measured by high resolution ultrasonic device (SONOS 5500, Hewlett Packard, U.S.A) under B-mode with 7.5 MHz probe (Hewlett Packard, U.S.A.).

For FMD measurement, the baseline BA diameter will be measured. Subsequently, a pneumatic cuff placed at the level of the left lower arm will be inflated to 250 mmHg for five minutes. After causing excessive blood flow in BA by wrist-cuff deflation, the BA diameter will be re-examined. FMD will be calculated as the percent maximum increase in arterial diameter. Arterial diameter will be defined as the distance between interface of intima and media and intima on opposite site. Measurement will be performed on endpoint of the dilation of heart(just before the R wave formation on ECG).

**3) Number of targeted subjects and randomization method : total 20 subjects**

Basis of calculation : In our preceding research upon healthy subjects, FMD before IR injury was 12.0 ± 6.2, and after IR was 4.6 ± 3.6. ^26^ Exenatide was proven to prevent IR injury 100 % (FMD before IR =15.0 ± 7.1, FMD after IR = 15.0 ± 5.9). Yet, the prevention effect of acupuncture has not been studied. We assumed that acupuncture could improve FMD after IR injury about 30%, as other researchers expect 30% effect in the same model.^27^ Targeted number of subjects is total 34(17 in each group), considering 5% type 1 error and 80% power. Considering drop-out rate of 15%, total 40 subjects are needed. As this study is crossover trial, 20 subjects are needed. We will use a permutable block design.

**4) Measuring method and clinical examination**

- Basic information includes research number, name, sex, age, medical history, family history, height(cm), weight(kg) .

- Blood plessure, ECG, FMD will be measured.

- Protection of privacy and research data : By encoding research data, no personal information will be included in the study result. Chief takes charge of security supervision of personal information before encoding.

**5) Statistical analysis**

All analyses will be performed with SPSS software(Windows version 17.0, Chicago, Illinois, USA). A p-value less than 0.05 will be considered statistically significant. Data will be presented as the mean ± standard deviation. Comparisons of continuous variable with normal distribution will be performed with student t-test or paired t-test. Nonparametric tests include Mann- Whitney test or Wilcoxon signed rank test. Categorical data will be analysed with Chi-square test. Change of FMD data between before and after IR injury will be analysed by two- way ANOVA.

6) Recruitment

Healthy volunteers will be recruited by recruitment announcement in Kyung Hee Medical Center and Kyung Hee University.

**9. Safety of participants**

As there will be no severe invasive treatment, we do not believe that there will be any serious side effects. In case of pain or cyanosis caused by 15minutes’ cuff pressure, medical team nearby will make a close observation. If necessary, pneumatic cuff will be removed immediately.

In complementary and alternative medicine, acupuncture is commonly used and it has been proven to be safe. Known adverse effects of acupuncture include local pain, bleeding, purpura, erythema, transient dizziness and nausea, which usually disappear in a few hours or days without any other medical interventions.

In case of light adverse effects, close observation by medical team will be provided until recovery. If severe side effects needing additional examination or treatment occur, expenses will be covered by research funds.

**10. Monitoring plan**

In order to guarantee subjects’ right and welfare, to check whether the clinical trial procedure is performed as documented and whether it obeys the relevant regulation, the principal investigator will actively cooperate with IRB for audit, monitoring and inspection.

**11. Research timetable**

| Months | 1 | 2 | 3 | 4 | 5 | 6 | 7 | 8 | 9 | 10 | 11 | 12 | 13-15 |
| --- | --- | --- | --- | --- | --- | --- | --- | --- | --- | --- | --- | --- | --- |
| Subjects enrollment |  |  |  |  |  |  |  |  |  |  |  |  |  |
| Measurement of endothelial cell |  |  |  |  |  |  |  |  |  |  |  |  |  |
| Data analysis |  |  |  |  |  |  |  |  |  |  |  |  |  |
| Paper work |  |  |  |  |  |  |  |  |  |  |  |  |  |

**12. References**

[1] Scheter M, Issachar A, Marai I, Koren-Morag N, Freinark D, Shahar Y, Shechter A, Feinberg MS. Long-term association of brachial artery flow-mediated vasodilation and cardiovascular events in middle-aged subjects with no apparent heart disease. Int J Cardiol 2009;134(1):52-8

[2] Simon A, Mijiti W, Gariepy J, Levenson J. Current possibilities for detecting high risk of cardiovascular disease. Int J Cardiol 2006;110(2):146

[3] Murohara T, Asahara T, Silver M, et al. Nitric oxide synthase modulates angiogenesis in response to tissue ischemia. J Clin Invest 1998;101:2567-78.

[5] Hill JM, Zalos G, Halcox JP, et al. Circulating endothelial progenitor cells, vascular function, and cardiovascular risk. N Engl J Med 2003;348:593-600.

[6] Gokce N, Keaney JF, Jr, Hunter LM, Watkins MT, Menzoian JO, Vita JA. Risk stratification for postoperative cardiovascular events via noninvasive assessment of endothelial function: a prospective study. Circulation 2002;105:1567-72.

[7] Widlansky ME, Gokce N, Keaney JF, Jr, Vita JA. The clinical implications of endothelial dysfunction. J Am Coll Cardiol 2003;42:1149-60.

[8] Cao Q, Liu J, Chen S, Han Z. Effects of electroacupuncture at neiguan on myocardial microcirculation in rabbits with acute myocardial ischemia. J Tradit Chin Med. 1998 Jun;18(2):134-9.

[9] Wu HC, Lin JG, Chu CH, Chang YH, Chang CG, Hsieh CL, Tsai AH, Ueng KC, Kuo WW, Lin JA, Liu JY, Huang CY. The effects of acupuncture on cardiac muscle cells and blood pressure in spontaneous hypertensive rats. Acupunct Electrother Res. 2004;29(1-2):83-95.

[10] Williams T, Mueller K, Cornwall MW. Effect of acupuncture-point stimulation on diastolic blood pressure in hypertensive subjects: a preliminary study. Phys Ther. 1991 Jul;71(7):523-9.

[11] Saku K, Mukaino Y, Ying H, Arakawa K. Characteristics of reactive electropermeable points on the auricles of coronary heart disease patients. Clin Cardiol. 1993 May;16(5):415-9.

[12] Tukmachi E, Jubb R, Dempsey E, Jones P. The effect of acupuncture on the symptoms of knee osteoarthritis--an open randomised controlled study. Acupunct Med. 2004 Mar;22(1):14-22.

[13] Park JM, Shin AS, Park SU, Sohn IS, Jung WS, Moon SK. The acute effect of acupuncture on endothelial dysfunction in patients with hypertension: a pilot, randomized, double-blind, placebo-controlled crossover trial. J Altern Complement Med. 2010 Aug;16(8):883-8.

[14] Kim DD, Pica AM, Durán RG, Durán WN. Acupuncture reduces experimental renovascular hypertension through mechanisms involving nitric oxide synthases. Microcirculation. 2006 Oct-Nov;13(7):577-85.

[15] Chen S, Ma SX. Nitric oxide in the gracile nucleus mediates depressor response to acupuncture (ST36). J Neurophysiol. 2003 Aug;90(2):780-5. Epub 2003 Apr 2.

[16] Li L, Yin-Xiang C, Hong X, Peng L, Da-Nian Z. Nitric oxide in vPAG mediates the depressor response to acupuncture in stress-induced hypertensive rats. Acupunct Electrother Res. 2001;26(3):165-70.

[18] Li P, Sun FY, Zhang AZ. The effect of acupuncture on blood pressure: the interrelation of sympathetic activity and endogenous opioid peptides. Acupunct Electrother Res. 1983;8(1):45-56.

[19] Mori H, Uchida S, Ohsawa H, Noguchi E, Kimura T, Nishijo K. Electro-acupuncture stimulation to a hindpaw and a hind leg produces different reflex responses in sympathoadrenal medullary function in anesthetized rats. J Auton Nerv Syst. 2000 Mar 15;79(2-3):93-8.

[20] Yao T. Acupuncture and somatic nerve stimulation: mechanism underlying effects on cardiovascular and renal activities. Scand J Rehabil Med Suppl. 1993;29:7-18.

[21] Michikami D, Kamiya A, Kawada T, Inagaki M, Shishido T, Yamamoto K, Ariumi H, Iwase S, Sugenoya J, Sunagawa K, Sugimachi M. Short-term electroacupuncture at Zusanli resets the arterial baroreflex neural arc toward lower sympathetic nerve activity. Am J Physiol Heart Circ Physiol. 2006 Jul;291(1):H318-26. Epub 2006 Feb 24.

[22] Laude K, Beauchamp P, Thuillez C, Richard V. Endothelial protective effects of preconditioning. Cardiovasc Res. 2002;55:466–473.

[23] Mankad PS, Amrani M, Rothery S, Severs NJ, Yacoub MH. Relative susceptibility of endothelium and myocardial cells to ischaemiareperfusion injury. Acta Physiol Scand. 1997;161:103–112.

[24] Tomai F, Crea F, Chiariello L, Gioffre PA. Ischemic preconditioning in humans: models, mediators, and clinical relevance. Circulation. 1999; 100:559–563.

[25] Beresewicz A, Maczewski M, Duda M. Effect of classic preconditioning and diazoxide on endothelial function and O2 _ and NO generation in thepost-ischemic guinea-pig heart. Cardiovasc Res. 2004;63:118–129.

[26] Ha SJ, Kim W, Woo JS, Kim JB, Kim SJ, Kim WS, Kim MK, Cheng XW, Kim KS. Preventive effects of exenatide on endothelial dysfunction induced by ischemia-reperfusion injury via KATP channels. Arterioscler Thromb Vasc Biol. 2012;32:474-480.

[27] Piot C, Croisille P, Staat P, Thibault H, Rioufol G, Mewton N, Elbelghiti R, Cung TT, Bonnefoy E, Angoulvant D, Macia C, Raczka F, Sportouch C, Gahide G, Finet G, André-Fouët X, Revel D, Kirkorian G, Monassier JP, Derumeaux G, Ovize M. Effect of cyclosporine on reperfusion injury in acute myocardial infarction. N Engl J Med. 2008;359:473-81.
